# Insect pollinators can unlock an annual monetary value of more than US $100 million from crop production in Rwanda

**DOI:** 10.1038/s41598-023-46936-w

**Published:** 2023-11-16

**Authors:** Rosita Endah Epse Yocgo, Isaac Hitimana, Malachie Hakizimana, Eliud Abucheli Birachi

**Affiliations:** 1 African Institute for Mathematical Sciences (AIMS) Rwanda, Kigali, Rwanda; 2 Institute for Plant Biotechnology, University of Stellenbosch, Stellenbosch, South Africa; 3 Pan Africa Bean Research Alliance- PABRA, International Center for Tropical Agriculture, CIAT, Kigali, Rwanda

**Keywords:** Ecology, Plant sciences, Environmental sciences

## Abstract

Insect pollinators provide a natural ecosystem service to more than 80% of known flowering plants, many of which are part of our diet. However, their importance in Africa and an agriculture-dependent country like Rwanda has yet to receive attention. This encumbers policy formulation and investments in insect pollinators as a strategic agronomic input. Meanwhile, Rwanda cultivates crops that can benefit significantly from insect pollinators for superior agronomic outputs. To uncover this, we characterized the dependence of the crop production subsector on insect pollinators. Using the bioeconomic approach, we assessed the total economic value and the value due to insect pollinators of crops cultivated in Rwanda. We also evaluated the crop's production value per ton and whether production would meet consumption demands in the complete absence of insect pollinators. Using 71 representative crops currently grown in Rwanda, we found a direct dependency of 62% on insect pollinators. Of 32 representative crops used for economic valuation in two years (2014 and 2020), their total monetary value is estimated at $2.551 billion to $2.788 billion. Direct insect pollinator-dependent crops accounted for 20% (2014) to 18% (2020) of this value, with the share attributed to insect pollinators above $100 million. The sector's vulnerability to insect pollinators decreased from 7.3% in 2014 to 4.3% in 2020. The mean production value per ton of the direct insect pollinator-dependent crops was found to be higher in 2014 before declining in 2020. Using 21 representative crops from 2014 to 2020, we found that many direct insect pollinator-dependent crops will struggle to meet consumption demands in the complete absence of all suitable insect pollinators. Finally, we propose interventions and future research that could be undertaken. These insights are a critical first step to propel the government to act on insect pollination to support its food security agenda.

## Introduction

Insect pollination is one of the imperative ecosystem services for more than 80% of known flowering plant species, many of which are part of our human diet^1,2^. These pollinators differentially improve crop quality/quantity and the availability of viable superior seeds^3^. Studies show that 75% of leading insect pollinator-dependent crops could account for 35% of global food production^4^. When the world was disaggregated by its economic regions, 59.8% of crops in developing regions were found to depend directly on these pollinators^5^. From a financial perspective, at the global scale, 51% (n = 46) of crops with a direct insect pollinator dependency accounted for 39% (625 billion euros) of the world's production value (€1618 trillion)^6^. The share from insect pollinators was estimated at €153 billion, while the agriculture sector's vulnerability to insect pollinator losses was about 9.8%^6^.

Food production constraints and economic losses due to declines of suitable insect pollinators could increase our dependence on already extensively consumed carbohydrate-dense staples (cereals, tubers, pulses), many of which are wind pollinated^5,7–9^. Insufficient consumption of a diverse number of nutrient-rich foods increases our vulnerability to non-communicable diseases^10–13^. Annual global deaths of up to 691,000 due to inadequate intake of insect-pollinator-dependent fruits, vegetables and nuts, which provide essential vitamins and oils, have been postulated from modelling studies^14^. Disease and death burdens would be higher in upper–middle–income countries^14^, while production losses would be higher in low-income countries, several of which are in Africa^14,15^.

More evidence is, however, still required to better establish the rate of species declines and extinction. This will allow scientists to fine-tune insect pollinator dependency studies and project high-risk crops. Nevertheless, a few global studies confirm that insect species, including taxa with insect pollinators like bees and butterflies, are declining faster than those increasing^16^. Model projections further depict more than 50% loss in suitable habitats globally for up to 49% of insect species and 44% of plant species if warming levels of 3.2 °C are attained^17^. Insect extinction risks of about 10% have also been predicted^18^, with the primary triggers being climate change, land use, pests, pathogens, and pollutants (pesticides and fertilizers)^19^. In Africa, most studies on insect declines are from farm-level studies on honey bees. Inputs like pesticides have been flagged as a threat to honey bee colonies in Rwanda^20^ and Kenya^21^. However, honeybees cannot account for the spatiotemporal behaviour of all species to stressors^18^.

As more tailored evidence on insect species decline is generated, global concerns and the existence of triggers that can affect plant-pollinator interactions^19,22^ are sufficient to drive preliminary impact assessment studies on crop-insect pollinator dependency. Results from the latter studies could provide feedback to impel insect pollinator monitoring actions. Unfortunately, in Africa and for several local crop varieties, insights on the percentage dependency of crops on insect pollinators are still lagging. While this remains a research gap, data can be inferred from available publications^4^ to support downstream studies. This has been the case in an increasing number of countries outside Africa, such as India^23^, Belgium^24^, Latin America^25^, China^26^, Pakistan^27^, and on a global scale^4,5^. However, only some studies have focused on African countries like Benin^28^, Burkina Faso^29^, Ethiopia^30^, Morroco^31^, Malawi, Nigeria, Tanzania, and Uganda^10^. Results show a high dependence of 46% (Malawi) to 76% (Tanzania).

To further exploit existing datasets and to expand knowledge on the dependency of the agriculture sector on insect pollinators in Africa, we elected to work in Rwanda, where a comprehensive study is yet to be undertaken. This is despite the country's high dependency on agriculture and the presence of crops like passion fruits, macadamias, mangoes, avocadoes, tomatoes, beans, eggplants, soybeans, coffee, groundnuts, etc., that can benefit from insect pollinators^32^. These crops are also essential for food security, health, nutrition and economic prosperity in Rwanda^33,34^. For instance, export revenues of up to $465.4M (2018/2019) have been generated by the sector, with marked contributions from coffee (~ $69M; insect-pollinated) and tea (~ $88M; wind-pollinated)^4,33^. More than half of the sector comprises household farmers^32,35^. In the Huye district of Rwanda, such farmers could lose up to 11% of farm income and 20% of marketed production share in the complete absence of insect pollinators^11^. Yet, like several African countries, Rwanda's Strategic Plan for Agriculture Transformation (PSTA4) 2018-202437 does not mention a crop-insect pollinator strategy. This, therefore, limits actions on and investments in insect pollination as an agronomic input.

This work thus aims to elucidate if the government of Rwanda should include insect pollinators as an agronomic input in its subsequent strategic plan for agriculture transformation. We hypothesized that insect pollinators are important for Rwanda's agriculture and would bring a valuable monetary contribution to the economy. Our study had the objective of mapping the insect pollinator dependency of 71 crops that are cultivated in Rwanda, evaluating the total economic value of crop production and that due to insect pollination for 32 representative crops using the bio-economic approach and FAO crop data for the year 2014 and 2020, and finally, evaluating whether production from 21 representative crops could meet consumption demands before and after the complete loss of insect pollinators. We found evidence for Rwanda to take strategic and urgent actions on insect pollination in its crop production subsector, including stringent insect pollinator conservation practices. Our findings also strengthen the need for other countries to commence such assessments and take action.

## Methodology

In this study, we assumed that all pollinators behave similarly (completely absent or completely present). This is irrespective of which crops they pollinate or how many pollinators can pollinate a given crop. Secondly, we used the mean pollinator dependency range/ratio of crops and not their actual pollinator dependency values. Data manipulation, analysis and visualization steps were performed using the R platform and suitable packages (Table_5_SuppInfo)^36^.

### Data extraction

Crop price and agriculture variables (yield, production and area harvested) data for the period 2010–2020 were downloaded from the Food and Agricultural Organization website^37^ in 2021 and then updated in February 2023, just before the completion of this study. These were downloaded as single files and then merged into one data set. Additional crops mentioned in the grey and published literature were used to enrich this data set. This included crops captured in the Agricultural Household Survey by the National Institute of Statistics of Rwanda^32^, the National Agricultural Export Development Board annual report^33^ and the Baseline Report on Rwanda Horticulture^38^. We extracted distinct crops from this data set, which resulted in a total of 71 representative crops. This data set was used to map the insect pollinator dependency of these crops. Crop type information was extracted from the Indicative Crop Classification (I.C.C.) list^39^.

### Crop-insect pollinator dependency mapping

The crop-insect pollinator dependency classification model by Klein et al.^4^ was used to classify the above crops. The insect pollinator dependency depicts the extent to which a plant depends on insect pollination for improved agronomic outputs (yields) or the production quantity the plant would lose in the complete absence of suitable insect pollinators. These dependencies are represented by lower and upper limit values between 0 and 100%, referred to as the insect pollinator dependency ratio, rate or range of a crop.

The model classifies plants into three main groups: direct, indirect, and none insect pollinator-dependent. Based on the above values, direct insect pollinator-dependent crops are further divided into four subgroups: essential (> 90–100%), great (> 40–90%), modest (> 10–40%), and little (> 0–10%). Indirect insect pollinator-dependent crops are divided into two subgroups: crops whose seed yields increase in the presence of insect pollinators and we consume their resultant vegetative parts (increase seed production, ISP), and crops whose seed production increases only during breeding, but we do not consume their resulting vegetative parts (increase breeding, IB). The latter crops reproduce through vegetative propagation. None insect pollinator-dependent crops do not require insect pollinators (wind-pollinated). Crops like tree tomatoes, stevia, moringa, and leeks with an unknown insect pollinator dependency were excluded.

The above classification model was further confirmed against country-specific crop dependency data sets from Brazil^40^, Argentina^41^, India^23^ and Burkina Faso^29^. Where discrepancies occurred, we used information from the most recent publications or the African-specific context (Table_1_SuppInfo). Stenchly et al.^29^ used a different classification for the indirect dependent group. They classified carrots, taro, onions and cabbages as directly dependent on insect pollinators, given that we consume their vegetative parts, whose production is influenced by seed quality/quantity achieved during insect pollination^42,43^.

The insect pollinator dependency table was generated after assigning an insect pollinator dependency (mean ratio, subgroup, group), crop name, species name, and crop type to each of the 71 crops in our data set (Table_1_SuppInfo). Crops with a different dependency value defined by Stenchly et al.^29^ were marked with an asterisk (*).

### Evaluation of the crops' distribution

The percentage distribution of a given pollinator-dependent group, subgroup or crop type was computed using data in the crop-insect pollinator-dependency table (Table_1_SuppInfo). Firstly, we aggregated (subaggregated) crops based on their pollinator-dependency groups (direct, indirect, none), subgroups (essential, great, modest, little, IB, ISP, none) or crop types (fruits, vegetables, nuts/oilseeds, stimulants, pulses, tubers, cereals, sugars, spices/herbs). For a given number of crops in a subaggregate (n_subaggregate_) and the total number of crops in the aggregate (n_aggregate_), the percentage distribution of the subaggregate is computed as in Eq. (1).

1 $${\text{Percentage distribution}}\;\left( \% \right) = 100\left( {n_{subaggregate} /n_{aggregate} } \right)$$

For instance, to compute the percentage distribution of the direct insect pollinator-dependent group, we divide the number of direct insect pollinator-dependent crops (n_subaggregate_) by the number of crops in the main aggregate/entire data set (n_aggregate_) and multiply by 100.

### Determination of the economic value of insect pollinators to crop production

The bioeconomic approach was applied to determine the economic/monetary value of insect pollinators to crop production^6^. It takes into account three input parameters: total crop production quantity (Q), farm gate price (P) and the insect pollinator dependency value (D). Hence, for a given crop (i), where i Є [1: I], the economic value (EV) is the product of the price (P_i_) and production quantity (Q_i_) as in Eq. (2). The economic value due to insect pollinators (IPEV) for a crop (i) is the product of its EV and the mean of its dependency ratio (D_i_) as in Eq. (3). The final results are expressed in the currency of the price.

2 $${\text{Economic value of crop production }}\left( {{\text{EV}}} \right) = \mathop \sum \limits_{i = 1}^{I} \left( {P_{i} *Q_{i} } \right)$$

3 $${\text{Economic value of insect pollination }}\left( {IPEV} \right) = \mathop \sum \limits_{i = 1}^{I} \left( {P_{i} *Q_{i} *D_{i} } \right)$$

$${Q}_{i}$$ = total agricultural production (tons) of a crop (*i*), $${P}_{i}$$($/tons) = the corresponding market value of the crop, $${D}_{i}$$= the crop's pollination dependence rate (%), which is represented by its mean dependency ratio. This includes essential crops (0.95), great (0.65), modest (0.25), and little (0.05), indirect and none pollinator-dependent crops (0.00) (Table_1_SuppInfo).

To calculate the EVs, we downloaded the FAO data set on the value of agricultural production from 2014 to 2020 as a single file. It contains crop data and the calculated economic value (U.S. dollars; $; Eq. 2) for several crops. We combined this with the data set on crop price, yield, production, and area harvested (Sect. 2.1) to easily compare different variables and obtain additional crop names, price and production data. Where applicable, we used the price and production information to calculate the EV of crops as outlined in Eq. (2). Crops lacking price and production data, or an EV, were eventually excluded, resulting in a final data set of 32 crops.

Using data in our crop insect pollinator-dependency table (Table_1_SuppInfo), we assigned an insect pollinator dependency (mean ratio, group and subgroup) primarily from Klein et al.^4^ as a new data set or from Stenchly et al.^29^, as a separate data set. We added the species name and crop type to these crops before calculating the IPEV (Eq. 3). All downstream steps were applied to both data sets. However, for visualization, the detailed outputs from the data set of the former authors are often displayed and described in the results.

For visualization, we aggregated crops based on their insect pollinator dependency group and crop type before computing their total EV, IPEV, area harvested and production for each year. Then, we displayed the results for 2014 and 2020 (Table_2_SuppInfo). The annual outputs mean and standard deviation for the entire study period (2014–2020) were also displayed based on their insect pollinator dependency groups when the classification model of Klein et al.^4^ (Table_3_SuppInfo) and Stenchly et al.^29^ (Table_4_SuppInfo) was applied.

### Determination of the vulnerability of the agricultural sector to insect pollinators

We calculated the vulnerability of the crop production subsector (CVR) if faced with a complete pollinator loss^6^ by dividing the IPEV sum by the EV sum for any crop category. The result is multiplied by 100 and expressed as a percentage, as in Eq. (4). In our study, the IPEV for indirect and wind-pollinated crops is zero when D_i_ = 0. Hence, this vulnerability solely depends on direct insect-pollinated crops when D_i_ > 0.

4 $${\text{Crop Vulnerability Ratio}}\;\left( {\% {\text{CVR}}} \right) = \frac{IPEV}{{EV}} = \frac{{\mathop \sum \nolimits_{i = 1}^{I} \left( {P_{i} *Q_{i} *D_{i} } \right)}}{{\mathop \sum \nolimits_{i = 1}^{I} \left( {P_{i} *Q_{i} } \right)}}*100$$

We computed the vulnerability ratio for each crop type and the annual vulnerabilities using the total economic values generated in Sect. 2.4 (Tables 2, 3, 4_SuppInfo).

### Determination of overproduction based on consumption demands

To understand whether production can meet consumption demands without insect pollinators, we used the food balance sheet data from FAO for Rwanda for 2014–2020^6^, which contains consumption and production data. Crops and their products (e.g. maize and its products) were first aligned to the main crop (maize). Processed foods (e.g. infant food), crop types (e.g. cereals and others) or miscellaneous were excluded as they do not represent a single crop. This resulted in a final data set of 21 crops. These crops were assigned an insect pollinator dependency (mean ratio, group and subgroup), species name and crop type as defined in our crop-insect pollinator dependency table (Table_1_SuppInfo).

For overproduction before pollination loss for a specific crop (i), where i Є [1: I], its consumption (C_i_) is subtracted from its total production quantity (Q_i_) and then divided by its consumption as in Eq. (5). For overproduction after pollination loss, we obtained the proportion of production that is not dependent on insect pollinators (1-D_i_), multiplied this proportion by its total production quantity (Qi), subtracted its consumption (C_i_) and divided the result by its consumption as in Eq. (6). These outputs were expressed as a percentage of consumption.

5 $${\text{Overproduction before pollination loss }}\left( {\% consumption} \right) = \frac{{\mathop \sum \nolimits_{i = 1}^{I} \left( {Q_{i} - C_{i} } \right)}}{{\mathop \sum \nolimits_{i = 1}^{I} \left( {C_{i} } \right)}}$$

6 $${\text{Overproduction after pollination loss }}\left( {\% consumption} \right) = \frac{{\mathop \sum \nolimits_{i = 1}^{I} \left( {Q_{i} \left( {1 - D_{i} } \right) - C_{i} } \right)}}{{\mathop \sum \nolimits_{i = 1}^{I} \left( {C_{i} } \right)}}$$

${\mathrm{Q}}_{\mathrm{i}}$ = total agriculture production (tons) of a given crop i, ${\mathrm{C}}_{\mathrm{i}}$ (tons) = consumption of crop i, ${\mathrm{D}}_{\mathrm{i}}$= the pollinator dependence rate of crop i (%), $(1-{\mathrm{D}}_{\mathrm{i}})$= portion of crop i without insect pollination.

### Determination of relative changes

Relative changes between two years of interest were calculated using the method described by Azien et al.^5^ and information in Table 2_SuppInfo. For this, one of the years was set as a reference year. Therefore, to compute the relative change in a variable (x) between two years, the value of a variable (x) in the reference year $(x_{{t_{reference} }} )$ is subtracted from that of the year of interest $(x_{t} )$ before dividing the output by the value of variable $(x)$ of the reference year $(x_{{t_{reference} }} )$ as in Eq. (7). In our study, 2014 was set as the reference year. Our variables included yield, production, area harvested, production value, economic value and consumption demand before or after the complete loss of insect pollination for any given crop. The final outputs are expressed as percentages.

7 $${\text{Relative percent change}}\;\left( {\% {\text{RC}}} \right) = \frac{{ (x_{{\dot{t}}} - x_{{t_{reference} }} )100}}{{x_{{t_{reference} }} }}$$

With $x_{{\dot{t}}}$ = value of a variable $(x)$ for a given crop in year t, $x_{treference}$ = value of variable $(x)$ for a given crop in the reference year.

### Missing data imputation

The Multivariate Imputation by Chained Equations (MICE) package in R was used to fill in missing data^44^. MICE has the advantage over other methods of filling in missing values many times based on the observed values of a given sample and the relationship in the data set^45^. The MICE imputation was used to fill the production value of green beans (2014 and 2015) and green chillies/peppers (2014).

### Statistical analysis

Statistical differences in production value between the three insect pollinator-dependent groups were calculated using the non-parametric Kruskal–Wallis test for each year. This is recommended for non-normally distributed data, especially if the researcher chooses not to manipulate the data through log transformation or removing outliers, for instance^46^. This test was selected over the analysis of variance (ANOVA) since the data for specific years (2014, 2015, 2016, 2020) arguably satisfied the test for normality, even though they satisfied the homogeneity of variance assumption. Secondly, not all crop types are represented in each insect pollinator-dependent group—this narrowed the number of independent variables that could be included.

The Kruskal–Wallis test was performed using its built-in function in RStudio. We defined two hypotheses, null and alternative, and then used the probability (p) value to statistically interpret the results^47^. For the null hypothesis, H_0_ implies that the means of the dependent variable (production value) is not significantly different (*p* > 0.05) among the independent variables (pollinator-dependent groups). Otherwise, we accept the alternative hypothesis H_1_ if at least one group mean differs significantly (*p* < 0.05) from the others. Lastly, the dunn_test function in RStudio was used to obtain information about which two combinations of the insect pollinator-dependent subgroups were statistically different.

## Results

### Dependence of edible crops on insect pollinators

#### Of the edible crops grown in Rwanda, 62% directly depend on insect pollinators

**Figure 1:**
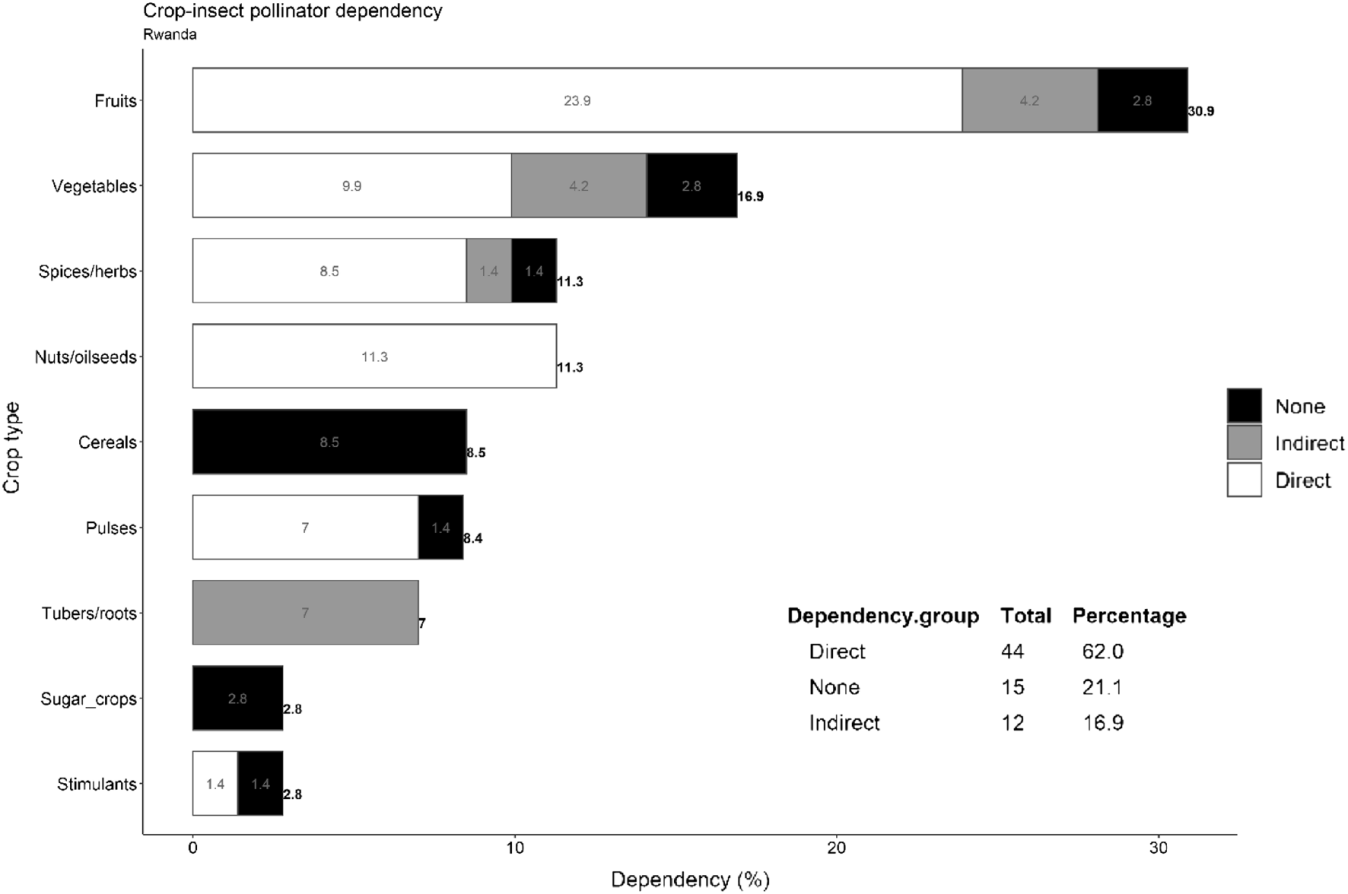
Dependence of 71 representative edible crops cultivated in Rwanda on insect pollinators. The crop-insect pollinator dependency ratio was inferred mainly from Klein et al.^4^ and other country-specific publications (Table_1_SuppInfo). The table in the chart depicts crops in each insect pollinator dependency group (direct, indirect, and none). The bar graph and associated digits represent the percentage distribution of each crop type.

We found that 62% (n = 44) of representative edible crops (n = 71) that are cultivated in Rwanda are directly dependent on insect pollinators (Fig. 1, table). Of these, the majority (14 crops) have a modest dependency, 12 crops a little dependency, 11 crops a great dependency and seven crops an essential dependency (Table_1_SuppInfo). Crops that did not require pollinators constituted the second largest group (21.1%, n = 15), followed by 16.9% (12 crops) with an indirect dependency. Among the latter group, the majority (n = 9) are pollinated by insects during crop breeding, whereas for the others (n = 4), insect pollination is vital for increased seed production.

#### Fruits, vegetables, and oilseeds substantially contribute to the direct insect pollinator dependency

The most representative crops were fruits, vegetables, nuts/oilseeds, and spices/herbs. The first three contributed the most to the overall dependence of the direct insect pollinator group. All represented nuts/oils have a direct dependency, while up to 77% (n = 17) of fruits and 58.3% (n = 7) of vegetables also have a direct dependency (Fig. 1, white bars). However, most nuts/oilseeds (75%) are at the lower end of the dependency grid of insect pollinators (modest – little), in contrast to 58.8% (n = 10) of fruits that occupy the higher end of the dependency grid (great-essential). For vegetables, there is a comparable distribution among the insect pollinator-dependent groups.

Among the least represented crop types, a majority have a direct dependency. We found that direct insect pollination is vital for 75% (n = 6) of spices/herbs. Of the six pulses, 83.4% occupy the lower end of the insect pollinator dependency grid. Of the two stimulants, there was an equal share, with coffee having a modest dependency and tea having no dependency. Tubers populated the indirect insect pollinator group, predominantly for breeding purposes. All representative sugar crops and cereals are wind-pollinated (Table_1_SuppInfo).

### Economic value of crop production and insect pollination

#### The total economic value of crop production increased in Rwanda from $2.551 billion (2014) to 2.788 billion (2020)

The total economic value of crop production from the 32 representative edible crops in Rwanda was in 2014 estimated at $ 2.551 billion (Table 1), with a production of 6.6 million tons across an estimated area of 2.1 million ha (Table_2_SuppInfo). Of this, the direct insect pollinator-dependent group had the highest number of representative crops (43.8%). This accounted for 20.4% ($521.5 million) of the economic value. Furthermore, its production (14.1% of the total) was slightly lower than that of the none-dependent group (15.7%). This former group also occupied the second-largest harvested area (31.8%).

**Table 1:** Total economic value of production (EV) in Rwanda in the year 2014 and 2020, share from insect pollination (IPEV), percentage relative change (RC) in the year 2020 against the reference year 2014, and vulnerability ratio (%VR).

	EV (USD)		IPEV (USD)			VR (%)		
	2014	2020	%RC	2014	2020	%RC	2014	2020
Direct								
Pulses	244.8 M	303.6 M	24.0	12.2 M	15.2 M	24.0	5.0	5.0
Vegetables	195.6 M	137.5 M	− 29.7	153.0 M	94.5 M	− 38.3	78.2	68.7
Nuts/oilseeds	28.7 M	39.3 M	36.8	4.1 M	4.9 M	21.6	14.1	12.6
Fruits	27.9 M	15.6 M	− 44.2	10.8 M	3.0 M	− 71.9	38.7	19.4
Stimulants	21.5 M	5.0 M	− 76.6	5.4 M	1.3 M	− 76.6	25.0	25.0
Spices/herbs	3.1 M	2.6 M	− 14.6	767.5 K	655.2 K	− 14.6	25.0	25.0
Total	521,498,000.00	503,546,000.00	–	186,242,200.00	119,515,500.00	–	–	–
Indirect								
Fruits	879.1 M	872.1 M	− 0.8	0.0	0.0	NaN	0.0	0.0
Tubers/roots	737.2 M	941.9 M	27.8	0.0	0.0	NaN	0.0	0.0
Vegetables	27.0 M	55.7 M	106.1	0.0	0.0	NaN	0.0	0.0
Total	1,643,315,000.00	1,869,746,000.00	–	0.00	0.00	–	–	–
None								
Cereals	346.0 M	354.8 M	2.6	0.0	0.0	NaN	0.0	0.0
Pulses	32.4 M	22.7 M	− 30.0	0.0	0.0	NaN	0.0	0.0
Stimulants	4.4 M	5.3 M	21.1	0.0	0.0	NaN	0.0	0.0
Sugar_crops	3.4 M	31.9 M	828.9	0.0	0.0	NaN	0.0	0.0
Total	386,180,000.00	414,668,000.00	–	0.00	0.00	–	–	–
Grand_total	2,550,993,000.00	2,787,960,000.00	–	186,242,200.00	119,515,500.00	–	–	–

Source: Own calculations using FAO_data.

M = million, K = thousand, USD = United States dollars.

Insect pollinator dependency values (D_i_) used to compute the IPEV were obtained primarily from Klein et al.^5^ (Table_1_SuppInfo).

The indirect insect pollinator-dependent group, which had 34.4% of representative crops, accounted for the highest total economic value (64.4%; $1.643 billion), production (70.2%), and harvested area (46.0%). The wind-pollinated group had the lowest number of representative crops (21.9%), made the lowest contribution to the economic value (15.1% = $386.2 million) and further occupied the smallest harvested area (22.2%).

The total economic value of crop production in 2020 increased by 9.3%, equating to $2.788 billion (Table 1). This was the second-highest economic value attained between 2014 and 2020 from representative crops (Table_3_SuppInfo). There was also a 33.1% increase in relative production (8.8 million tons) and a 5.8% increase in harvested area (2.2 million ha), despite the pressures imposed by COVID-19. Additionally, the economic value of the direct insect pollinator-dependent group decreased slightly by 3.4%, resulting in an estimated value of 503.5 million. Nevertheless, this was still higher than that of the none-dependent group ($414.6 million; relative increase = 7.3%) but lower than that of the indirect dependent group, which still made the highest contribution of 58.5% ($1.869 billion; relative increase = 13.8%).

The direct insect pollinator group had a 4.5% increase in production (966,356 tons) and a 25% increase in its area harvested (826,475 ha) in 2020. Its production remained slightly less than that of the non-insect pollinator-dependent group (1 million tons) and far less than that of the indirect-dependent group (Table_2_SuppInfo). This latter group accounted for up to 77.5% (6.8 million tons) of the overall production, even though its area harvested had declined by 15% (811,063 ha).

At the crop type level, indirect insect pollinator-dependent fruits and tubers contributed the most to the total economic value (Table 1). The respective amounts were $879.1 million and $737.2 million in 2014, before decreasing slightly in fruits (0.8%) but increasing in 2020 by 27.8% in tubers. Cereals, pulses (direct) and vegetables (direct) followed suit, with 130–354 million.

#### The economic value of crops due to insect pollination declined by 35.8% in 2020

**Figure 2:**
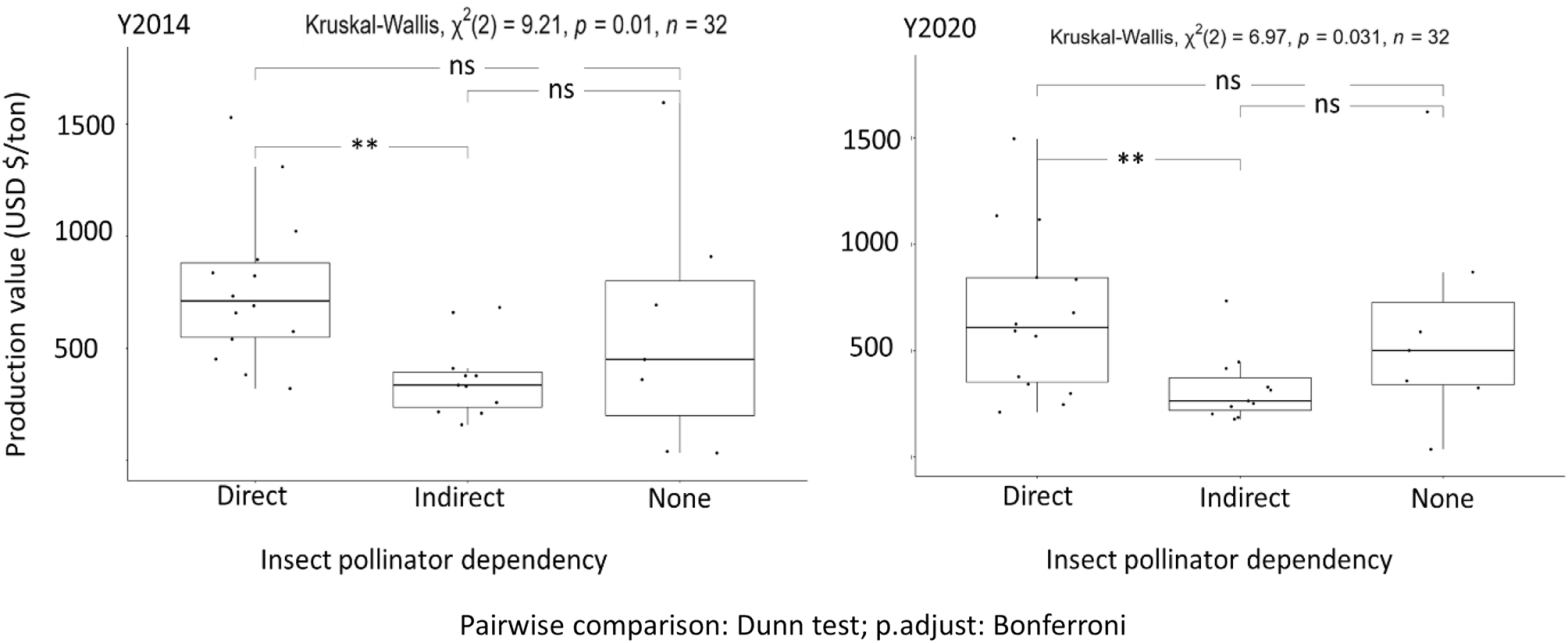
Production value per ton ($/ton) of representative crops cultivated in Rwanda in 2014 and 2020. The values are represented with respect to their insect pollinator-dependent group (direct, indirect, and none). Grey dots represent the mean production value per ton in each group. Kruskal–Wallis and Dunn tests were used to calculate significant differences between groups using their functions in RStudio.

The share of the total economic value of production due to insect pollination was in 2014 at an estimated $186.2 million (Table 1). This translated into a 7.3% vulnerability of the crop production subsector to insect pollination. However, this share declined in 2020 by 35.8% (Table 1), with a reduced vulnerability to insect pollinators of 4.3%. Stimulants (76.6%), fruits (71.9%) and vegetables (38.3%), represented mainly by coffee, mangoes, and pumpkins, contributed to this decline (Figure_2_SuppInfo).

Amongst the crop types, vegetables (direct) accounted for the highest economic value due to insect pollination in both study years (Table 1). This value was in 2014, $153 million, before declining by 38.3% ($94.5 million) in 2020. They were also the most vulnerable crop type in 2014 (78.2%) and 2020 (68.7%). The pulses had an estimated value of 12.2–15.2 million. Unlike vegetables, pulses were the least vulnerable to insect pollinators (5%). Fruits, estimated at $10.8 million in 2014, were the second most vulnerable crop type (38.7%). They also experienced one of the highest declines (71.9%) in 2020. The stimulant, coffee, was the fourth highest contributor in 2014 ($5.4 million) but experienced a 76.6% decline in 2020, resulting in nuts/oilseeds taking this spot. Stimulants also had the same vulnerability (25%) as spices, which made the least contribution in both years (Table 1).

When we recalculated the IPEV using the classification model of Stenchly et al.^29^, the total monetary value was estimated at $219.91 million in 2014 and increased to $235.24 in 2020. The sector's vulnerability was estimated at 8.62% in 2014 and 8.44% in 2020 (Table 4_SuppInfo).

#### The insect pollinator-dependent group had the lowest production per hectare of harvested area but the highest production value per ton

We also observed that in terms of production quantity per hectare of harvested area, the direct insect pollinator-dependent crops were producing, on average, the lowest quantity per hectare (977,737.69 tons/733,687.5 ha = 1.33 tons/ha) over the 7-year (2014–2020) period (Table 3_SuppInfo). The wind-pollinated group had a slightly higher production per hectare of harvested area (934,986.5 tons /514,047.43 ha = 1.82 tons/ha), while the indirect dependency group generated the highest quantity (5,509,515.06 tons/875,269.86 ha = 6.29 tons/ha).

The direct insect pollinator-dependent group had the highest mean production value per ton in 2014 and 2020. In 2014, this value was 769.9 $$\pm$$ 91.3$/ton (± standard error) and was significantly greater (*p* = 0.025) than that of the indirect dependent group (366.2 $$\pm$$ 51.5$/ton) but not the none-dependent group (584.0 $$\pm$$ 207.5 $/ton; *p* = 0.18). This value decreased in 2020 by 13.0% and 11.5% in the direct and indirect insect pollinator-dependent groups, respectively, but increased slightly (5.2%) in the none-dependent group (Fig. 2). The value in 2020 remained significantly different (*p* = 0.010) only between the direct and indirect insect pollinator-dependent groups. Dry peas (none) had the highest production value/ton in both years, followed by groundnuts, coffee, lemon, oranges, mangoes (direct dependencies) and rice (none) in at least one of these years.

### Production and consumption demands

#### Insect pollinator-dependent crops could face major constraints in meeting consumption demands

Using data from 2014 to 2020, we found that among the eight representative direct insect pollinator-dependent crops, only coffee, and in selected study years, chillies, groundnuts and dry beans could meet consumption demands (Figure_2_SuppInfo). In groundnuts, this was apparent during the later years of the study (2017–2020), while in chillies, this was between 2015 and 2019. In contrast, oranges, soybeans, lemon/limes, and tomatoes had differential deficits ranging from 30 to 70% during the study period. The latter was affected after the loss of insect pollinators, whereas the others were affected under both situations. On average, over the seven years, oranges and soybeans showed deficits during both pollination situations, while the others had marked deficits (tomatoes and oranges) or declines (chillies and dry beans) only after pollinator loss (Table 2).

**Table 2:** Mean overproduction from 2014 to 2020 before and after insect pollinator loss (%consumption).

	Crop_type	Overproduction (% consumption)	
		Before. pollinator.loss	After. pollinator.loss
Direct			
Coffee, green, modest	Stimulants	935.8 ± 213.2	676.8 ± 159.9
Chillies and peppers, green, modest	Spices/herbs	36.4 ± 7	2.3 ± 5.3
Groundnuts, little	Nuts/oilseeds	19 ± 8.6	13.1 ± 8.1
Beans, dry, little	Pulses	7.1 ± 2.2	1.7 ± 2.1
Tomatoes, great	Vegetables	6.7 ± 0.7	− 62.6 ± 0.2
Oranges, little	Fruits	− 41.3 ± 4	− 44.2 ± 3.8
Lemons and limes, little	Fruits	1 ± 1	− 4 ± 1
Soya beans, modest	Nuts/oilseeds	− 0.5 ± 4.1	− 25.4 ± 3.1
Indirect			
Bananas, increased breeding	Fruits	37.8 ± 0	N.A
Cassava, fresh, increased breeding	Tubers/roots	0.3 ± 1.9	N.A
Onions, shallots dry, increased seed production	Vegetables	− 0.5 ± 2.1	N.A
Pineapples, increased breeding	Fruits	7.7 ± 1	N.A
Plantains, cooking bananas, increased breeding	Fruits	9.1 ± 0	N.A
Potatoes, increased breeding	Tubers/roots	8 ± 16.6	NA
Sweet potatoes, increased breeding	Tubers/roots	17 ± 2.7	N.A
Yams, increased breeding	Tubers/roots	27 ± 12.2	N.A
None			
Maize (corn), none	Cereals	55.8 ± 10	N.A
Peas, dry, none	Pulses	11.7 ± 4	N.A
Rice, none	Cereals	− 30.6 ± 3.1	N.A
Sorghum , None	Cereals	25.2 ± 3	NA
Wheat, None	Cereals	− 88 ± 1.5	N.A

Source: Own calculations using FAO_data.

Of the eight indirect insect pollinator-dependent crops, only potatoes, onions, and cassava could not meet consumption demands (Figure_3_SuppInfo and Table 2). While this was marginal in onions and cassava (± 10%), it was pronounced in potatoes during the early study years (2014–2018), with deficits of at least 40%, before recovering to exceed consumption demands (10%) during the later years.

Wheat and rice were the two wind-pollinated crops that could not meet consumption demands (Table 2), with deficits of at least 30% (Figure_4_SuppInfo). Although dry peas initially had a slight deficit (~ − 10%), they recovered in the subsequent years, remaining at marginal levels (~ 10–15%). Maize and sorghum, on the other hand, remained in excess of consumption demands throughout the study period, with excesses of about 30–100% observed.

## Discussion

Our study has provided for the first time a more advanced insight into the dependence of the crop production subsector in Rwanda on insect pollinators, the economic value of crop production and the impact on consumption demands before and after the complete loss of suitable insect pollinators. What is unique in our study is the multiyear approach applied to observe fluctuations, if any, using the most recent data.

In our study, we found support for our hypothesis that insect pollinators are important to Rwanda's agriculture and will bring in a valuable monetary contribution. This was despite the absence of the added economic value of 30 insect-pollinated crops (e.g. passion fruits, macadamia, sunflowers, etc.) with no price data^6^. Losing this monetary contribution has repercussions on farmers' revenue^11^ and GDP^48^.

A loss of more than 60% of insect-pollinated crops in Rwanda due to a complete absence of these pollinators would affect household farmers who cultivate fruits (with 67.3% growing avocadoes and 32.7% mangoes), vegetables (with 43.7% growing amaranth, 25.6% tomato, 24.2% eggplant), pulses (88% growing beans), nuts/oilseed (12.3% growing soybean, 6% growing groundnuts) and stimulants (355, 771 farmers)^32,49^. Production losses will compound food scarcity, shift consumption demands to indirect- or wind-pollinated staples (maize, rice, cassava, beans, etc.), and heightened food insecurity, especially in resource-limited households^50^.

Even though food choices are not guided by pollination, farming households in Rwanda (80%) rely heavily on their farm produce for consumption^10,32^. This includes insect-pollinated produce, especially vitamin A-rich vegetables (pumpkin, carrot, squash, amaranth/dodo), fruits (passion fruit, avocado, papaya), but also nuts/oilseed (groundnuts), pulses (dried beans), spices/herbs and coffee^34,50^. Additionally, their consumption, which increases with availability, can be enhanced by promoting kitchen gardens and capacity-building programs on food and nutrition^13,51^. These actions align with the government of Rwanda's strategic plan to sustain a nutrition-sensitive agriculture sector which increases dietary diversity and the consumption of nutrient-dense foods^34,35^. This continues to be important in combating the 35% food insecurity risk, 33% stunting, 45% mortality risk (especially among children under five years), 26% obesity in women, and inadequate consumption of diverse crop types in Rwanda^34,52,53^.

A focused intervention should, therefore, be on insect-pollinated vegetables and fruits, which, in our study, were the most vulnerable to insect pollinator losses and the highest contributors to the vulnerability of the production subsector. The latter vulnerability has remained slightly lower than that for world agriculture^4^ but is higher in given years than for East Africa^6^. Together with nuts/oilseeds (soybean and groundnuts), these vegetables (tomatoes) and fruits (oranges, lemon) are among the crops that struggled to meet consumption demands in the 7-year study period.

Low-vulnerability crops (nuts/oilseeds, pulses, coffee) should also be given attention due to their added nutritional value (oils, proteins, vitamins) and international traction (coffee)^14^. To close nutrition gaps, biofortification is used to augment the nutrient composition of consumer-preferred crops (e.g. dry beans for iron delivery) in Rwanda^9^ and globally^54^. However, if insect pollination is vital to these biofortified crops, appropriate interventions should be prescribed to achieve superior yields. For instance, dry beans can further benefit from yield increases per hectare of up to 46% during open pollination by insects^55^.

Our findings are consistent with postulations that low-income countries are yet to reap the full benefits of insect pollination^5,14,15^. Our representative direct insect pollinator-dependent group generated the least production per hectare of area harvested. Given that agricultural land is a limited resource^37^, Rwanda might struggle to compete in production quantity. However, they can capitalize on improving quality through strategic insect pollinator interventions as an added agronomic input. This will better position their produce in the international market.

## Conclusion and recommendations

Given the potential value-addition of crop-insect pollination to food systems, nutrition and health, Rwanda should incorporate relevant insect pollination actions in its strategic plans.

A first recommendation is for Rwanda to increase efforts on beehive establishment, mainly where pollinator-dependent crops are cultivated. This will directly benefit several vital crops like coffee, sunflower, macadamia, mango, avocado, etc., that depend on bees and other pollinators^4^.

Secondly, policies guiding bee hive establishment should align with those regulating pollutive fertilizers, herbicides and pesticides^56^. While these are strategic farm inputs in Rwanda^35^, their negative consequences on bees and other pollinators are a concern^20^. Insect-friendly farm inputs (organic fertilizers) should be promoted, while pesticides should be carefully analyzed for their impacts on insects and discontinued if harmful.

Thirdly, Rwanda should nurture natural ecosystems, which are pollinator-rich habitats^57,58^. Insect-pollinated crops could be cultivated close to such vicinities to facilitate interactions. Additionally, Rwanda's land consolidation action^35^ should promote the establishment of patches of fields with high pollinator attractants (e.g. sunflower or flowering plants with bright colours) close to areas with pollinator-dependent crops^58,59^.

Above all, advocacy and capacity-building activities should be intensified^35^. Additional emphasis should be on the adequate handling of approved farm inputs, techniques to mitigate the impacts of harmful farm practices, the importance of pollinators in food systems, monitoring and documenting insect visitors, hand pollination techniques, and climate change mitigation actions (e.g. afforestation)^60^.

## Challenges and future perspectives

This study has also identified gaps, mainly around the availability of data. Research efforts should be geared towards characterizing the insect pollinator dependency of crop cultivars in Rwanda, their nutritional value, and possible health benefits. This will open the gateway for impact-driven crop pollinator studies. A second research effort should focus on monitoring insect pollinator species in different ecosystems to clarify concerns on whether, how and at what rate insect pollinators are declining. Thirdly, national surveys and data collection efforts should capture crop production, area cultivated, area harvested, yield, farm gate price, and consumption demand data more consistently for an increasing number of crops, including minority crops in Rwanda. This data should also be disaggregated per crop and not aggregated under a crop type. These findings and recommendations could contribute to Rwanda's poverty alleviation and food security agenda, and could be translated in other African countries.

### Supplementary Information

Supplementary Information 1.

Supplementary Information 2.

## Data Availability

This published article and its supplementary information files include all data generated or analyzed during this study.

## References

[1] Ollerton J, Winfree R, Tarrant S (2011). How many flowering plants are pollinated by animals?. Oikos.

[2] Rodger JG (2021). Widespread vulnerability of flowering plant seed production to pollinator declines. Sci. Adv..

[3] McGregor SE (1976). Insect Pollination of Cultivated Crop Plants.

[4] Klein A-M (2007). Importance of pollinators in changing landscapes for world crops. Proc. R. Soc. B Biol. Sci..

[5] Aizen MA, Garibaldi LA, Cunningham SA, Klein AM (2009). How much does agriculture depend on pollinators? Lessons from long-term trends in crop production. Ann. Bot..

[6] Gallai N, Salles J-M, Settele J, Vaissière BE (2009). Economic valuation of the vulnerability of world agriculture confronted with pollinator decline. Ecol. Econ..

[7] Ranum P, Peña-Rosas JP, Garcia-Casal MN (2014). Global maize production, utilization, and consumption. Ann. N. Y. Acad. Sci..

[8] Singh N, Vasudev S, Yadava DK, Chaudhary DP, Prabhu KV, Chaudhary DP, Kumar S, Langyan S (2014). Oil Improvement in maize: Potential and prospects. Maize: Nutrition Dynamics and Novel Uses.

[9] McDermott J, Wyatt AJ (2017). The role of pulses in sustainable and healthy food systems: Pulses in food systems. Ann. N. Y. Acad. Sci..

[10] Tibesigwa, B. *Naturally Available Pollinator Decline Will Decrease Household Food and Increase Gender-Gap in Nutrition between Men and Women Who Head Smallholder Farm Households in Sub-Saharan Africa* (2018).

[11] Uwingabire, Z. *Evaluating the impacts of pollinators decline on social welfare at different spatial scales: Economic and nutritional aspects* (Université Toulouse le Mirail-Toulouse II, 2021).

[12] Carazo A (2021). Vitamin A update: Forms, sources, kinetics, detection, function, deficiency, therapeutic use and toxicity. Nutrients.

[13] Sly BC (2023). Increasing household diet diversity and food security in rural Rwanda using small-scale nutrition-sensitive agriculture: A community-engaged proof-of-concept study. Nutrients.

[14] Smith MR (2022). Pollinator deficits, food consumption, and consequences for human health: A modeling study. Environ. Health Perspect..

[15] Murphy JT, Breeze TD, Willcox B, Kavanagh S, Stout JC (2022). Globalisation and pollinators: Pollinator declines are an economic threat to global food systems. People Nat..

[16] Sánchez-Bayo F, Wyckhuys KAG (2021). Further evidence for a global decline of the entomofauna. Austral Entomol..

[17] Warren R, Price J, Graham E, Forstenhaeusler N, VanDerWal J (2018). The projected effect on insects, vertebrates, and plants of limiting global warming to 1.5 °C rather than 2 °C. Science.

[18] Watson, R. & Baste, I. *Summary for Policymakers of the Global Assessment Report on Biodiversity and Ecosystem Services of the Intergovernmental Science-Policy Platform on Biodiversity and Ecosystem Services, IPBES Secretariat, Bonn, Germany* 22–47 (2019).

[19] Dicks, L. V. *et al.* A global assessment of drivers and risks associated with pollinator decline. *Res. Square* 1–21 (2020).

[20] Alexandre NP (2020). Effect of farming practices on honey production in boundary of Gishwati Forest National Park. J. Geosci. Environ. Prot..

[21] Kanda, R. K. *Effect of Pesticides Use on Honeybee (Apis mellifera L.) Mortality and Honey Production in Transmara West Sub-county, Narok County, Kenya* (Maseno University, 2016).

[22] Memmott J, Craze PG, Waser NM, Price MV (2007). Global warming and the disruption of plant–pollinator interactions. Ecol. Lett..

[23] Chaudhary OP, Chand R (2017). Economic benefits of animal pollination to Indian agriculture. Indian J. Agric. Sci..

[24] Jacquemin F, Violle C, Rasmont P, Dufrêne M (2017). Mapping the dependency of crops on pollinators in Belgium. One Ecosyst..

[25] Basualdo M (2022). Current status and economic value of insect-pollinated dependent crops in Latin America. Ecol. Econ..

[26] An J, Chen W (2011). Economic value of insect pollination for fruits and vegetables in China. Acta Entomol. Sin..

[27] Irshad M, Stephen E (2013). Value of insect pollinators to agriculture of Pakistan. Int. J. Agron. Agric. Res..

[28] Toni H, Djossa BA (2015). Economic value of pollination services on crops in Benin, West Africa. Int. J. Biol. Chem. Sci..

[29] Stenchly K, Hansen MV, Stein K, Buerkert A, Loewenstein W (2018). Income vulnerability of West African farming households to losses in pollination services: A case study from Ouagadougou, Burkina Faso. Sustainability.

[30] Alebachew GW (2018). Economic value of pollination service of agricultural crops in Ethiopia: Biological pollinators. J. Apic. Sci..

[31] Sabbahi R (2022). Economic value of insect pollination of major crops in Morocco. Int. J. Trop. Insect Sci..

[32] NISR. *Agricultural Household Survey 2020, Annual Report. National Institute of Statistics of Rwanda.* https://www.statistics.gov.rw/publication/agricultural-household-survey-2020 (2022).

[33] NAEB. *Agriculture Export Performance. National Agricultural Export Development Board, NAEB 2021–2022*. https://naeb.gov.rw/fileadmin/Reports-Annual/ANNUAL_REPORT_2021-2022.pdf (2021).

[34] National Institute of Statistics of Rwanda - NISR, Ministry of Health - MOH & ICF. *Rwanda demographic and health survey 2019–2020. Kigali, Rwanda and Rockville, Maryland, USA: NISR/MOH/ICF.* https://dhsprogram.com/pubs/pdf/FR370/FR370.pdf (2021).

[35] Ministry of Agriculture and Animal Resources (MINAGRI). *PSTA 4—Rwanda Strategic Plan for Agriculture Transformation 2018–24*. https://www.minagri.gov.rw/fileadmin/user_upload/Minagri/Publications/Policies_and_strategies/PSTA4__Rwanda_Strategic_Plan_for_Agriculture_Transformation_2018.pdf (2018).

[36] R version 4.2.2 Development Core Team. A language and environment for statistical computing. R Foundation for Statistical Computing https://www.R-project.org/. (2022).

[37] FAO, F. A. O. *Statistical databases. Food and Agriculture Organization of the United Nations.* http://faostat.fao.org/ (2023).

[38] Clay, D. *et al.* Baseline report on the Rwanda horticulture Organisations survey. *AGRER Consort.* 92 (2014).

[39] FAO. *World Programme for the Census of Agriculture 2020*. (2015).

[40] Giannini TC, Cordeiro GD, Freitas BM, Saraiva AM, Imperatriz-Fonseca VL (2015). The dependence of crops for pollinators and the economic value of pollination in Brazil. J. Econ. Entomol..

[41] Chacoff, N. P., Morales, C. L., Garibaldi, L. A., Ashworth, L. & Aizen, M. A. Pollinator dependence of Argentinean agriculture: Current status and temporal analysis (2010).

[42] Grubben G (2014). Vegetables to combat the hidden hunger in Africa. Chron. Hortic..

[43] Negi N (2020). Role of pollinators in vegetable seed production. J. Entomol. Zool. Stud..

[44] Van Buuren S, Groothuis-Oudshoorn K (2011). mice: Multivariate imputation by chained equations in R. J. Stat. Softw..

[45] Azur MJ, Stuart EA, Frangakis C, Leaf PJ (2011). Multiple imputation by chained equations: What is it and how does it work?. Int. J. Methods Psychiatr. Res..

[46] Smalheiser NR, Smalheiser NR (2017). Chapter 12—Nonparametric tests. Data Literacy.

[47] Andrade C (2019). The P value and statistical significance: Misunderstandings, explanations, challenges, and alternatives. Indian J. Psychol. Med..

[48] Statistical Yearbook. *National Institute of Statistics of Rwanda*. https://www.statistics.gov.rw/publication/1767 (2021).

[49] NAEB. *NAEB strategic plan 2019–2024. Increasing Agri-export revenues.* https://naeb.gov.rw/fileadmin/documents/STRATEGIC%20PLAN.pdf (2019).

[50] Weatherspoon DD, Miller S, Ngabitsinze JC, Weatherspoon LJ, Oehmke JF (2019). Stunting, food security, markets and food policy in Rwanda. BMC Public Health.

[51] Habimana O, Muhawenayo J (2023). Can kitchen gardens improve household food security? Causal evidence in a large sample from Rwanda. SSRN Electron. J..

[52] Luo H, Zyba SJ, Webb P (2020). Measuring malnutrition in all its forms: An update of the net state of nutrition index to track the global burden of malnutrition at country level. Glob. Food Secur..

[53] Nyiraneza, L., Matsiko, E., Chantal, G. & Nahimana, R. Double burden of malnutrition in Rwanda: Systematic review of Rwandan Demographic health Survey, 2005, 2010 and 2015 (2018).

[54] Bouis HE, Saltzman A (2017). Improving nutrition through biofortification: A review of evidence from HarvestPlus, 2003 through 2016. Glob. Food Secur..

[55] Elisante F (2020). Insect pollination is important in a smallholder bean farming system. PeerJ.

[56] Ndakidemi B, Mtei K, Ndakidemi PA (2016). Impacts of synthetic and botanical pesticides on beneficial insects. Agric. Sci..

[57] Martins, D. J. People, plants and pollinators: uniting conservation, food security, and sustainable agriculture in East Africa. *Conserv. Biol. Voices Trop.* 232–238 (2013).

[58] Proesmans W, Bonte D, Smagghe G, Meeus I, Verheyen K (2019). Importance of forest fragments as pollinator habitat varies with season and guild. Basic Appl. Ecol..

[59] Ratto F (2021). Proximity to natural habitat and flower plantings increases insect populations and pollination services in South African apple orchards. J. Appl. Ecol..

[60] Mutabazi, A. Assessment of operational framework related to climate change in Rwanda. In: *Rwanda Environment Management Authority, Kigali, Rwanda* (2010).

