# Supplementary material for: Insect pollinators can unlock an annual monetary value of more than US $100 million from crop production in Rwanda
Source: Sci Rep. 2023 Nov 16;13:20108. doi: 10.1038/s41598-023-46936-w (PMC10654601; doi:10.1038/s41598-023-46936-w)
Supplement: Supplementary file 2 — Supplementary Information 2. [file 41598_2023_46936_MOESM2_ESM.docx]

Supplementary tables and figures

*Table_1*_SuppInfo*: Crop-insect pollinator dependency table*

*Rwanda*

|  | *Species* | *Crop* | *Mean. Di (%)* | *Reference.Di* |
| --- | --- | --- | --- | --- |
| Direct - Essential | | | | |
| Fruits | *Annona cherimola, A. senegalensis* | Cherimoya, custard apple | 0.95 | *in Klein et al., 2007* |
| Fruits | *Passiflora edulis* | Passion fruit | 0.95 | *in Klein et al., 2007* |
| Fruits | *Citrullus lanatus* | Watermelons | 0.95 | *in Klein et al., 2007* |
| Nuts/oilseeds | *Macadamia ternifolia* | Macadamia nut | 0.95 | *in Klein et al., 2007* |
| Spices/herbs | *Vanilla spp.* | Vanilla | 0.95 | *in Klein et al., 2007* |
| Vegetables | *Cleome gynandra* | African spider flower | 0.95 | *in Stenchly et al., 2018* |
| Vegetables | *Cucurbita spp. Lagenaria siceraria* | Pumpkins, squash and gourds | 0.95 | *in Klein et al., 2007* |
| Direct - Great | | | | |
| Fruits | *Malus domestica* | Apples | 0.65 | *in Klein et al., 2007* |
| Fruits | *Persea americana* | Avocados | 0.65 | *in Klein et al., 2007* |
| Fruits | *Jatropha curcas(L.)* | Barbados nut/jatropha | 0.65 | *in Chaudhary and Chand, 2017* |
| Fruits | *Psidium guajava* | Guava | 0.65 | *in Giannini et al., 2015* |
| Fruits | *Prunus salicina* | Japanese plum | 0.65 | *in Klein et al., 2007* |
| Fruits | *Mangifera indica, Garcinia mangostana, Psidium guajava* | Mangoes, mangosteens, guavas | 0.65 | *in Klein et al., 2007* |
| Fruits | *Prunus domestica, P. spinosa* | Plums and sloes | 0.65 | *in Jacquemin et al., 2017* |
| Nuts/oilseeds | *Helianthus annus* | Sunflower | 0.65 | *in Giannini et al., 2015* |
| Spices/herbs | *Apium graveolens* | Celery | 0.65 | *in Chaudhary and Chand, 2017* |
| Vegetables | *Cucumis sativus* | Cucumbers and gherkins | 0.65 | *in Klein et al., 2007* |
| Vegetables | *Solanum lycopersicum* | Tomatoes | 0.65 | *in Giannini et al., 2015, in Chaudhary and Chand, 2017* |
| Direct - Modest | | | | |
| Fruits | *Physalis peruviana* | Cape gooseberries | 0.25 | *in Jacquemin et al., 2017* |
| Fruits | *Ficus carica* | Fig | 0.25 | *in Klein et al., 2007* |
| Fruits | *Punica granatum* | Pomegranate | 0.25 | *in Klein et al., 2007* |
| Fruits | *Fragaria spp.* | Strawberries | 0.25 | *in Klein et al., 2007* |
| Nuts/oilseeds | *Brassica carinata* | Ethiopian mustard | 0.25 | *in Klein et al., 2007* |
| Nuts/oilseeds | *Brassica napus* | Rapeseed | 0.25 | *in Klein et al., 2007* |
| Nuts/oilseeds | *Gossypium hirsutum* | Seed cotton | 0.25 | *in Klein et al., 2007* |
| Nuts/oilseeds | *Glycine max* | Soybeans | 0.25 | *in Klein et al., 2007* |
| Pulses | *Dolichos biflorus, D. lablab* | Hyacinth bean, lablab | 0.25 | *in Klein et al., 2007* |
| Spices/herbs | *Capsicum annuum Group* | Chillies and peppers, green | 0.25 | *in Chaudhary and Chand, 2017* |
| Spices/herbs | *Petroselinum crispum* | Parsley | 0.25 | *in Chaudhary and Chand, 2017, in Stenchly et al., 2018* |
| Stimulants | *Coffea spp.* | Coffee, green | 0.25 | *in Klein et al., 2007* |
| Vegetables | *Solanum melongena* | Eggplants (aubergines) | 0.25 | *in Klein et al., 2007* |
| Vegetables | *Abelmoschus esculentus* | Okra | 0.25 | *in Klein et al., 2007* |
| Direct - Little | | | | |
| Fruits | *Citrus limon, C. aurantiifolia* | Lemons and limes | 0.05 | *in Klein et al., 2007* |
| Fruits | *Citrus x sinensis* | Oranges | 0.05 | *in Klein et al., 2007* |
| Fruits | *Carica papaya* | Papayas | 0.05 | *in Klein et al., 2007* |
| Nuts/oilseeds | *Arachis hypogaea* | Groundnuts, with shell | 0.05 | *in Klein et al., 2007* |
| Nuts/oilseeds | *Elaeis guineensis* | Oil palm fruit | 0.05 | *in Klein et al., 2007* |
| Pulses | *Phaseolus vulgaris* | Beans, dry | 0.05 | *in Klein et al., 2007* |
| Pulses | *Vigna sp., Phaseolus sp.* | Beans, green | 0.05 | *in Klein et al., 2007* |
| Pulses | *Vigna unguiiculata* | Cowpea | 0.05 | *in Klein et al., 2007* |
| Pulses | *Cajanus cajan* | Pigeon pea | 0.05 | *in Klein et al., 2007* |
| Spices/herbs | *Piper spp.* | Pepper (piper spp.) | 0.05 | *in Chaudhary and Chand, 2017* |
| Spices/herbs | *Capsicum annuum* | Sweet pepper | 0.05 | *in Klein et al., 2007* |
| Vegetables | *Amaranths spp.* | Amaranthus | 0.05 | *in Stenchly et al., 2018* |
| Indirect - Increased breeding | | | | |
| Fruits | *Musa spp.* | Bananas | Indirect | *in Klein et al., 2007* |
| Fruits | *Ananas comosus* | Pineapples | Indirect | *in Klein et al., 2007* |
| Fruits | *Musa spp.* | Plantains and others | Indirect | *in Klein et al., 2007* |
| Spices/herbs | *Allium sativum* | Garlic | Indirect | *in Klein et al., 2007* |
| Tubers/roots | *Manihot esculenta* | Cassava | Indirect | *in Klein et al., 2007* |
| Tubers/roots | *Solanum tuberosum* | Potatoes | Indirect | *in Klein et al., 2007* |
| Tubers/roots | *Ipomoea batatas* | Sweet potatoes | Indirect | *in Klein et al., 2007* |
| Tubers/roots | *Dioscorea alata* | Yams | Indirect | *in Klein et al., 2007* |
| Indirect - Increased seed production | | | | |
| Tubers/roots | *Colocasia esculenta* | Taro (cocoyam)* | Indirect | *in Klein et al., 2007* |
| Vegetables | *Brassica oleracea var. capitata* | Cabbages and other brassicas* | Indirect | *in Klein et al., 2007* |
| Vegetables | *Daucus carota, Brassica rapa subsp. rapa* | Carrots and turnips* | Indirect | *in Klein et al., 2007* |
| Vegetables | *Allium cepa* | Onions, dry* | Indirect | *in Klein et al., 2007* |
| None | | | | |
| Cereals | *Hordeum vulgare* | Barley | 0 | *in Klein et al., 2007* |
| Cereals | *Zea mays* | Maize | 0 | *in Klein et al., 2007* |
| Cereals | *Pennisetum glaucum* | Millet | 0 | *in Klein et al., 2007* |
| Cereals | *Oryza sativa* | Rice, paddy | 0 | *in Klein et al., 2007* |
| Cereals | *Sorghum bicolor* | Sorghum | 0 | *in Klein et al., 2007* |
| Cereals | *Triticum aestivum* | Wheat | 0 | *in Klein et al., 2007* |
| Fruits | *Vitis vinifera* | Grapes | 0 | *in Klein et al., 2007* |
| Fruits | *Olea europaea* | Olives | 0 | *in Chaudhary and Chand, 2017* |
| Pulses | *Pisum sativum* | Peas, dry | 0 | *in Klein et al., 2007* |
| Spices/herbs | *Zingiber officinale* | Ginger | 0 | *in Chaudhary and Chand, 2017* |
| Stimulants | *Camellia sinensis* | Tea | 0 | *in Klein et al., 2007* |
| Sugar_crops | *Beta vulgaris* | Sugar beet | 0 | *in Klein et al., 2007* |
| Sugar_crops | *Saccharum officinarum* | Sugar cane | 0 | *in Klein et al., 2007* |
| Vegetables | *Agaricus bisporus* | Mushrooms and truffles | 0 | *in Chaudhary and Chand, 2017* |
| Vegetables | *Spinacia Oleracea* | Spinach | 0 | *in Klein et al., 2007* |
| *Source: Own calculations using FAO_data* | | | | |
| **The insect pollinator classification of Stenchly et al., 2018 categorizes these crops as directly dependent on insect pollinators with mean dependency ratio (Di) = 0.95 (essential) for carrots and taro, 0.65 (great) for onions and 0.05 (little) for cabbage.* | | | | |
| *Reference: Refer to References.* | | | | |

Table_2_SuppInfo: Total economic value of production (EV), share due to insect pollination (IPEV), relative change (RC, %) against year 2014, and agronomic parameters.

|  | EV (USD) | |  | IPEV (USD) | | Production (tons) | |  | Area. harvested (ha) | |  |
| --- | --- | --- | --- | --- | --- | --- | --- | --- | --- | --- | --- |
|  | 2014 | 2020 | %RC | 2014 | 2020 | 2014 | 2020 | %RC | 2014 | 2020 | %RC |
| Mangoes, guavas, mangosteens,  Great | 13.207M | 1.798M | -86.4 | 8.585M | 1.169M | 12.906K | 2.148K | -83.4 | 3.879K | 175.000 | -95.5 |
| Lemons and limes, Little | 5.921M | 8.020M | 35.5 | 296.050K | 401.000K | 7.073K | 7.069K | -0.1 | 1.497K | 1.445K | -3.5 |
| Oranges, Little | 3.369M | 2.931M | -13.0 | 168.450K | 146.550K | 3.758K | 2.626K | -30.1 | 2.060K | 1.353K | -34.3 |
| Papayas, Little | 2.936M | 865.000K | -70.5 | 146.800K | 43.250K | 4.458K | 2.278K | -48.9 | 229.000 | 107.000 | -53.3 |
| Avocados, Great | 2.443M | 1.947M | -20.3 | 1.588M | 1.266M | 7.600K | 6.477K | -14.8 | 650.000 | 293.000 | -54.9 |
| Subtotal fruits | 27,876,000.00 | 15,561,000.00 | — | 10,783,800.00 | 3,025,050.00 | 35,794.99 | 20,598.88 | — | 8,315.00 | 3,373.00 | — |
| Groundnuts, Little | 15.576M | 24.389M | 56.6 | 778.800K | 1.219M | 10.181K | 16.304K | 60.1 | 27.513K | 35.901K | 30.5 |
| Soya beans, Modest | 13.133M | 14.885M | 13.3 | 3.283M | 3.721M | 17.901K | 23.755K | 32.7 | 37.728K | 48.488K | 28.5 |
| Subtotal nuts/oilseeds | 28,709,000.00 | 39,274,000.00 | — | 4,062,050.00 | 4,940,700.00 | 28,082.00 | 40,058.35 | — | 65,241.00 | 84,389.00 | — |
| Beans, dry, Little | 239.175M | 297.453M | 24.4 | 11.959M | 14.873M | 415.259K | 438.736K | 5.7 | 465.865K | 647.983K | 39.1 |
| Beans, green, Little | 5.628M | 6.154M | 9.3 | 281.400K | 307.700K | 6.832K | 7.282K | 6.6 | 912.000 | 951.000 | 4.3 |
| Subtotal pulses | 244,803,000.00 | 303,607,000.00 | — | 12,240,150.00 | 15,180,350.00 | 422,091.11 | 446,017.77 | — | 466,777.00 | 648,934.00 | — |
| Chillies and peppers, green, Modest | 3.070M | 2.621M | -14.6 | 767.500K | 655.250K | 4.443K | 4.595K | 3.4 | 323.000 | 1.315K | 307.1 |
| Subtotal spices | 3,070,000.00 | 2,621,000.00 | — | 767,500.00 | 655,250.00 | 4,443.36 | 4,595.48 | — | 323.00 | 1,315.00 | — |
| Coffee, green, Modest | 21.456M | 5.021M | -76.6 | 5.364M | 1.255M | 16.379K | 20.459K | 24.9 | 55.030K | 17.208K | -68.7 |
| Subtotal stimulants | 21,456,000.00 | 5,021,000.00 | — | 5,364,000.00 | 1,255,250.00 | 16,379.00 | 20,459.00 | — | 55,030.00 | 17,208.00 | — |
| Pumpkins, squash, gourds, Essential | 112.413M | 55.234M | -50.9 | 106.792M | 52.472M | 248.285K | 260.931K | 5.1 | 48.267K | 50.613K | 4.9 |
| Tomatoes, Great | 63.599M | 53.574M | -15.8 | 41.339M | 34.823M | 117.415K | 90.509K | -22.9 | 8.545K | 12.930K | 51.3 |
| Eggplants (aubergines), Modest | 19.572M | 28.654M | 46.4 | 4.893M | 7.163M | 51.154K | 83.186K | 62.6 | 6.699K | 7.713K | 15.1 |
| Subtotal vegetables | 195,584,000.00 | 137,462,000.00 | — | 153,024,700.00 | 94,458,900.00 | 416,852.88 | 434,626.50 | — | 63,511.00 | 71,256.00 | — |
| Total, Direct | 521,498,000 | 503,546,000.00 |  | 186,242,200.00 | 119,515,500.00 | 923,643.3 | 966,355.98 |  | 659,197 | 826,475.00 |  |
| Bananas, IB | 709.799M | 637.390M | -10.2 | NA | NA | 1.039M | 2.032M | 95.6 | 409.960K | 178.983K | -56.3 |
| Plantains, cooking bananas, IB | 161.921M | 229.961M | 42.0 | NA | NA | 765.629K | 913.231K | 19.3 | 222.961K | 106.409K | -52.3 |
| Pineapples, IB | 7.398M | 4.727M | -36.1 | NA | NA | 19.538K | 17.829K | -8.7 | 2.000K | 3.579K | 79.0 |
| Subtotal fruits | 879,118,000.00 | 872,078,000.00 | — | 0.00 | 0.00 | 1,824,187.29 | 2,963,133.02 | — | 634,921.00 | 288,971.00 | — |
| Cassava, fresh, IB | 303.271M | 306.330M | 1.0 | NA | NA | 900.227K | 1.280M | 42.1 | 68.852K | 192.156K | 179.1 |
| Sweet potatoes, IB | 204.691M | 256.384M | 25.3 | NA | NA | 940.786K | 1.276M | 35.6 | 140.977K | 179.555K | 27.4 |
| Potatoes, IB | 186.299M | 283.334M | 52.1 | NA | NA | 719.006K | 858.521K | 19.4 | 68.208K | 104.494K | 53.2 |
| *Taro, ISP | 22.167M | 84.340M | 280.5 | NA | NA | 66.879K | 188.042K | 181.2 | 24.924K | 29.015K | 16.4 |
| Yams, IB | 20.727M | 11.554M | -44.3 | NA | NA | 54.718K | 61.733K | 12.8 | 6.140K | 5.951K | -3.1 |
| Subtotal tubers | 737,155,000.00 | 941,942,000.00 | — | 0.00 | 0.00 | 2,681,616.00 | 3,663,491.81 | — | 309,101.00 | 511,171.00 | — |
| *Cabbages, IB | 11.351M | 10.869M | -4.2 | NA | NA | 71.135K | 61.245K | -13.9 | 5.595K | 3.729K | -33.4 |
| *Onions, shallots_dry, ISP | 9.556M | 25.177M | 163.5 | NA | NA | 14.463K | 34.297K | 137.1 | 2.408K | 3.629K | 50.7 |
| *Carrots, turnips, ISP | 6.135M | 19.680M | 220.8 | NA | NA | 14.916K | 47.335K | 217.3 | 1.682K | 3.563K | 111.8 |
| Subtotal vegetables | 27,042,000.00 | 55,726,000.00 | — | 0.00 | 0.00 | 100,513.91 | 142,876.72 | — | 9,685.00 | 10,921.00 | — |
| Total, Indirect | 1,643,315,000.00 | 1,869,746,000.00 |  | 0.00 | 0.00 | 4,606,317.20 | 6,769,501.55 |  | 953,707.00 | 811,063.00 |  |
| Maize (corn), None | 210.854M | 160.501M | -23.9 | 0.000 | 0.000 | 583.096K | 448.633K | -23.1 | 233.150K | 294.439K | 26.3 |
| Rice, None | 66.182M | 101.169M | 52.9 | 0.000 | 0.000 | 72.723K | 116.504K | 60.2 | 23.770K | 29.584K | 24.5 |
| Sorghum, None | 63.465M | 85.603M | 34.9 | 0.000 | 0.000 | 140.578K | 170.489K | 21.3 | 137.227K | 169.419K | 23.5 |
| Wheat, None | 5.472M | 7.530M | 37.6 | 0.000 | 0.000 | 7.886K | 12.811K | 62.5 | 4.204K | 12.309K | 192.8 |
| Subtotal cereals | 345,973,000.00 | 354,803,000.00 | — | 0.00 | 0.00 | 804,283.00 | 748,437.55 | — | 398,351.00 | 505,751.00 | — |
| Peas, dry, None | 32.367M | 22.656M | -30.0 | 0.000 | 0.000 | 20.276K | 13.953K | -31.2 | 37.082K | 17.261K | -53.5 |
| Tea leaves, None | 4.409M | 5.340M | 21.1 | 0.000 | 0.000 | 108.000K | 140.005K | 29.6 | 17.220K | 22.348K | 29.8 |
| Sugar cane, None | 3.431M | 31.869M | 828.9 | 0.000 | 0.000 | 101.080K | 98.283K | -2.8 | 6.168K | 8.309K | 34.7 |
| Total, None | 386,180,000.00 | 414,668,000.00 |  |  |  | 1,033,639.18 | 1,000,679.00 |  | 458,821.00 | 553,669.00 |  |
| Grand_total | 2,550,993,000.00 | 2,787,960,000.00 | — | 186,242,200.00 | 119,515,500.00 | 6,563,599.72 | 8,736,536.53 | — | 2,071,725.00 | 2,191,207.00 | — |
| Source: Own calculations using FAO_data | | | | | | | | | | | |
| Cell colours: Five highest contributors per variable, mean vulnerability ratio and totals, differentiated by varying grey colours | | | | | | | | | | | |
| EV: total economic value, IPEV: Economic value due to insect pollination, IB: Increased breeding, ISP: Increased seed production | | | | | | | | | | | |
| M: million, K: thousand, ha: hectare, USD = United States Dollar | | | | | | | | | | | |
| *The insect pollinator classification of Stenchly et al., 2018 categorizes these crops as directly dependent on insect pollinators with mean dependency ratio (Di) = 0.95 (essential) for carrots and taro, 0.65 (great) for onions and 0.05 (little) for cabbage.  With this classification, the IPEV for carrots = $5,828,250 (2014) and $18,696,000 (2020), taro = $21,058,650 (2014) and $80,123,000 (2020), onions = $6211400 (2014) and $16,365,050 (2020), cabbages = $567,550 (2014) and $543,450 (2020).  How this classification further influences the annual area harvested, production, yield, total economic value, total economic value due to insect pollinators and the vulnerability is shown in Table 4_SuppInfo. | | | | | | | | | | | |

*Table 3_SuppInfo: Agronomic parameters, the total economic value of production (EV), share due to insect pollination (IPEV)and vulnerability ratio (VR) from 2014 to 2020 for crops cultivated in Rwanda based on their insect pollinator-dependent groups depicted primarily by Klein et al., 2007.*

|  | Area harvested (ha) | Production (tons) | Yield (hg/ha) | EV (USD) | IPEV (USD) | VR (%) |
| --- | --- | --- | --- | --- | --- | --- |
| Direct insect pollinator-dependent | | | | | |  |
| 2014 | 659,197 | 923,643.3 | 908,051 | 521,498,000 | 186,242,200 |  |
| 2015 | 685,230 | 944,757.2 | 880,992 | 498,772,000 | 180,862,200 |  |
| 2016 | 694,488 | 936,531.5 | 799,276 | 453,100,000 | 141,081,900 |  |
| 2017 | 752,879 | 977,032.4 | 632,798 | 457,826,000 | 134,587,100 |  |
| 2018 | 747,174 | 1,055,111.7 | 1,495,313 | 439,270,000 | 131,644,000 |  |
| 2019 | 770,370 | 1,040,731.7 | 969,817 | 470,772,000 | 125,547,800 |  |
| 2020 | 826,475 | 966,356.0 | 993,702 | 503,546,000 | 119,515,500 |  |
| *Total* | *5,135,813.00* | *6,844,163.80* | *6,679,949.00* | *3,344,784,000.00* | *1,019,480,700.00* |  |
| *Mean* | *733,687.57* | *977,737.69* | *954,278.43* | *477,826,285.71* | *145,640,100.00* |  |
| *Standard deviation* | *57,646.75* | *51,310.96* | *267,518.65* | *30,428,046.35* | *26,814,669.21* |  |
| Indirect insect pollinator-dependent | | | | | |  |
| 2014 | 953,707 | 4,606,317.2 | 852,088 | 1,643,315,000 | 0 |  |
| 2015 | 979,593 | 4,712,511.8 | 806,941 | 1,703,254,000 | 0 |  |
| 2016 | 1,023,952 | 4,801,181.5 | 708,726 | 1,736,433,000 | 0 |  |
| 2017 | 809,511 | 5,842,868.2 | 816,609 | 2,309,108,000 | 0 |  |
| 2018 | 769,487 | 5,362,104.8 | 876,586 | 1,687,00,3000 | 0 |  |
| 2019 | 779,576 | 6,472,120.4 | 1,080,193 | 1,709,490,000 | 0 |  |
| 2020 | 811,063 | 6,769,501.5 | 1,029,104 | 1,869,746,000 | 0 |  |
| *Total* | *6,126,889.00* | *38,566,605.40* | *6,170,247.00* | *12,658,349,000.00* | *0.00* |  |
| *Mean* | *875,269.86* | *5,509,515.06* | *881,463.86* | *1,808,335,571.43* | *0.00* |  |
| *Standard deviation* | *106,408.14* | *875,570.87* | *130,240.67* | *231,833,349.36* | *0.00* |  |
| None insect pollinator-dependent | | | | | |  |
| 2014 | 458,821 | 1,033,639.2 | 316,658 | 386,180,000 | 0 |  |
| 2015 | 469,677 | 838,402.5 | 299,991 | 310,603,000 | 0 |  |
| 2016 | 497,115 | 880,204.0 | 231,705 | 341,386,000 | 0 |  |
| 2017 | 529,501 | 862,328.5 | 264,340 | 388,786,000 | 0 |  |
| 2018 | 539,779 | 954,598.5 | 267,527 | 336,885,000 | 0 |  |
| 2019 | 549,770 | 975,053.8 | 270,475 | 398,212,000 | 0 |  |
| 2020 | 553,669 | 1,000,679.0 | 264,106 | 414,668,000 | 0 |  |
| *Total* | *3,598,332.00* | *6,544,905.50* | *1,914,802.00* | *2,576,720,000.00* | *0.00* |  |
| *Mean* | *514,047.43* | *934,986.50* | *273,543.14* | *368,102,857.14* | *0.00* |  |
| *Standard deviation* | *38,842.20* | *74,885.09* | *27,471.73* | *38,349,331.02* | *0.00* |  |
| *The sum of all insect pollinator-dependent groups for each year* | | | | | | |
| *2014* | *2,071,725* | *6,563,600* | *2,076,797* | *2,550,993,000* | *186,242,200* | *7.30* |
| *2015* | *2,134,500* | *6,495,672* | *1,987,924* | *2,512,629,000* | *180,862,200* | *7.20* |
| *2016* | *2,215,555* | *6,617,917* | *1,739,707* | *2,530,919,000* | *141,081,900* | *5.57* |
| *2017* | *2,091,891* | *7,682,229* | *1,713,747* | *3,155,720,000* | *134,587,100* | *4.26* |
| *2018* | *2,056,440* | *7,371,815* | *2,639,426* | *2,463,158,000* | *131,644,000* | *5.34* |
| *2019* | *2,099,716* | *8,487,906* | *2,320,485* | *2,578,474,000* | *125,547,800* | *4.87* |
| *2020* | *2,191,207* | *8,736,537* | *2,286,912* | *2,787,960,000* | *119,515,500* | *4.29* |
| *Source: Own calculations using FAO_data* | | | | | |  |
| *Cell colour: Years with the highest output in each insect pollinator-dependent group* | | | | | |  |
| *EV: total economic value, IPEV: Economic value due to insect pollination, ha: hectare, hg: hectogram* | | | | | |  |
| *The insect pollinator classification (Table 1_SuppInfo), primarily from Klein et al., 2007 was used to calculate the IPEV. In this classification, the mean dependency value (Di) for carrots, taro, onions and cabbage = 0 and are considered as indirect insect pollinator-dependent crops. Their IPEVs therefore = 0* | | | | | |  |

Table 4_SuppInfo: Agronomic parameters, the total economic value of production (EV), share due to insect pollination (IPEV)and vulnerability ratio (VR) from 2014 to 2020 for crops cultivated in Rwanda based on their insect pollinator-dependent groups depicted primarily by Stenchly et al., 2018.

|  | Area harvested (ha) | Production (tons) | Yield (hg/ha) | EV (USD) | IPEV (USD) | VR (%) | |
| --- | --- | --- | --- | --- | --- | --- | --- |
| Direct | | | | | |  | |
| 2014 | 693,806 | 1,091,036.2 | 1,210,753 | 570,707,000 | 219,908,050 |  | |
| 2015 | 722,851 | 1,112,241.9 | 1,184,955 | 551,904,000 | 218,807,200 |  | |
| 2016 | 742,777 | 1,169,318.6 | 1,076,045 | 553,211,000 | 225,417,350 |  | |
| 2017 | 820,988 | 1,313,165.3 | 901,166 | 606,071,000 | 258,227,150 |  | |
| 2018 | 787,229 | 1,348,486.3 | 1,880,797 | 547,860,000 | 216,488,300 |  | |
| 2019 | 807,857 | 1,343,460.6 | 1,451,671 | 593,300,000 | 225,190,800 |  | |
| 2020 | 866,411 | 1,297,274.5 | 1,450,103 | 643,612,000 | 235,243,000 |  | |
| *Total* | *5,441,919.00* | *8,674,983.50* | *9,155,490.00* | *4,066,665,000.00* | *1,599,281,850.00* |  | |
| *Mean* | *777,417.00* | *1,239,283.36* | *1,307,927.14* | *580,952,142.86* | *228,468,835.71* |  | |
| *Standard deviation* | *60,565.96* | *111,513.45* | *319,594.81* | *35,409,750.87* | *14,500,983.57* |  | |
| Indirect | | | | | |  | |
| 2014 | 919,098 | 4,438,924.3 | 549,386 | 1,594,106,000 | 0 |  | |
| 2015 | 941,972 | 4,545,027.2 | 502,978 | 1,650,122,000 | 0 |  | |
| 2016 | 975,663 | 4,568,394.3 | 431,957 | 1,636,322,000 | 0 |  | |
| 2017 | 741,402 | 5,506,735.2 | 548,241 | 2,160,863,000 | 0 |  | |
| 2018 | 729,432 | 5,068,730.2 | 491,102 | 1,578,413,000 | 0 |  | |
| 2019 | 742,089 | 6,169,391.5 | 598,339 | 1,586,962,000 | 0 |  | |
| 2020 | 771,127 | 6,438583.1 | 572,703 | 1,729,680,000 | 0 |  | |
| *Total* | *5,820,783.00* | *36,735,785.70* | *3,694,706.00* | *11,936,468,000.00* | *0.00* |  | |
| *Mean* | *831,540.43* | *5,247,969.39* | *527,815.14* | *1,705,209,714.29* | *0.00* |  | |
| *Standard deviation* | *108,654.79* | *814,060.81* | *56,309.52* | *207,526,242.97* | *0.00* |  | |
| None | | | | | |  | |
| 2014 | 458,821 | 1,033,639.2 | 316,658 | 386,180,000 | 0 |  | |
| 2015 | 469,677 | 838,402.5 | 299,991 | 310,603,000 | 0 |  | |
| 2016 | 497,115 | 880,204.0 | 231,705 | 341,386,000 | 0 |  | |
| 2017 | 529,501 | 862,328.5 | 264,340 | 388,786,000 | 0 |  | |
| 2018 | 539,779 | 954,598.5 | 267,527 | 336,885,000 | 0 |  | |
| 2019 | 549,770 | 975,053.8 | 270,475 | 398,212,000 | 0 |  | |
| 2020 | 553,669 | 1,000,679.0 | 264,106 | 414,668,000 | 0 |  | |
| *Total* | *3,598,332.00* | *6,544,905.50* | *1,914,802.00* | *2,576,720,000.00* | *0.00* |  | |
| *Mean* | *514,047.43* | *934,986.50* | *273,543.14* | *368,102,857.14* | *0.00* |  | |
| *Standard deviation* | *38,842.20* | *74,885.09* | *27,471.73* | *38,349,331.02* | *0.00* |  | |
| *The sum of all insect pollinator-dependent groups for each year* | | | | | | | |
| *2014* | *2,071,725* | *6,563,600* | *2,076,797* | *2,550,993,000* | *219,908,050* | *8.62* | |
| *2015* | *2,134,500* | *6,495,672* | *1,987,924* | *2,512,629,000* | *218,807,200* | *8.71* | |
| *2016* | *2,215,555* | *6,617,917* | *1,739,707* | *2,530,919,000* | *225,417,350* | *8.91* | |
| *2017* | *2,091,891* | *7,682,229* | *1,713,747* | *3,155,720,000* | *258,227,150* | *8.183* | |
| *2018* | *2,056,440* | *7,371,815* | *2,639,426* | *2,463,158,000* | *216,488,300* | *8.79* | |
| *2019* | *2,099,716* | *8,487,906* | *2,320,485* | *2,578,474,000* | *225,190,800* | *8.73* | |
| *2020* | *2,191,207* | *8,736,537* | *2,286,912* | *2,787,960,000* | *235,243,000* | *8.44* | |
| *Source: Own calculations using FAO_data* | | | | | | |  |
| *Cell colour: Years with the highest output in each insect pollinator-dependent group* | | | | | | |  |
| *EV: total economic value, IPEV: Economic value due to insect pollination, ha: hectare, hg: hectogram* | | | | | | |  |
| *The insect pollinator classification (Table 1_SuppInfo*), primarily from Stenchly et al., 2018 was used to calculate the IPEV. In this classification, the mean dependency value (Di) for carrots = 0.95, taro = 0.95, onions = 0.65 and cabbage = 0.05 and are considered as direct insect pollinator-dependent crops.* | | | | | | |  |

Table_5_SuppInfo: R packages and libraries used.

| R packages/libraries | Function |
| --- | --- |
| gt | Data visualization |
| dplyr | Data manipulation |
| tidyverse | Data importation, tidying, manipulation, and visualization |
| formattable | Data visualization |
| ggpubr | Data visualization and statistical analysis |
| webshot2 | Data visualization |
| highcharter | Data visualization |
| viridisLite | Assessing colors |
| colorspace | Assessing colors |
| cowplot | Data visualization |
| gridExtra | Data visualization, arranging plots |
| scales | Data visualization |
| ggplot2 | Data visualization |
| mice | Missing data imputation |


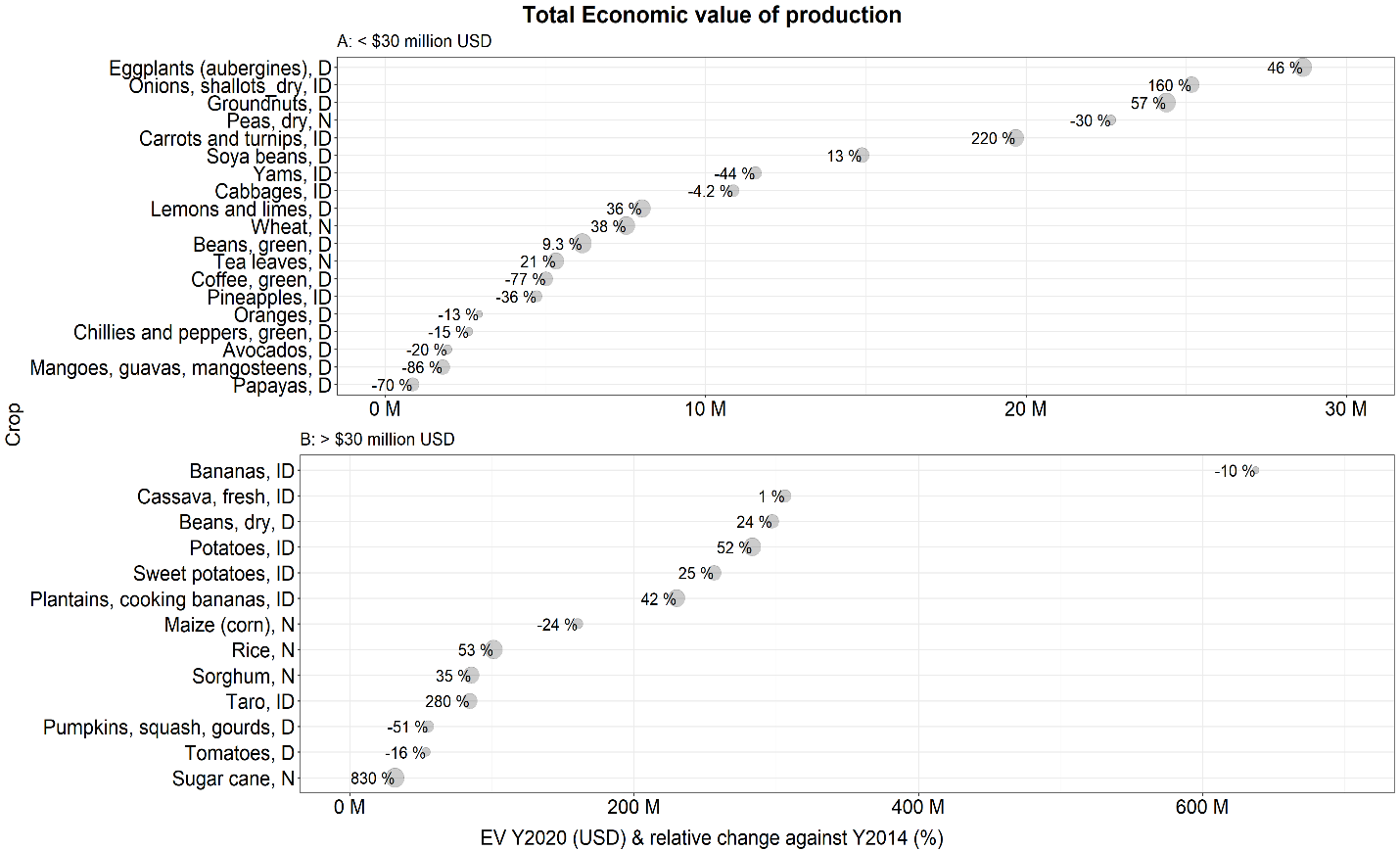


*Figure_1*_*SuppInfo: Total economic value and relative changes in crops cultivated in Rwanda between 2014 and 2020. The bioeconomic approach* (Gallai et al., 2009) *was used to evaluate the economic value of 31 crops* (FAO, 2023) *cultivated in Rwanda and for which production and production value data existed for 2014 and 2020. The relative change against the reference year 2014 was calculated and represented as percentages inside the plot. The x-axis represents the total monetary value in millions (M). Each crop was assigned an insect pollinator dependency group (D: direct, ID: indirect or N: none) based on their demand for insect pollination. Data analysis and visualization were done using the R platform* (R version 4.2.2 Development Core Team, 2022) *and suitable packages. Plot A and B represent crops that respectively contributed under 50 million USD or above 50 million USD to the total economic value.*


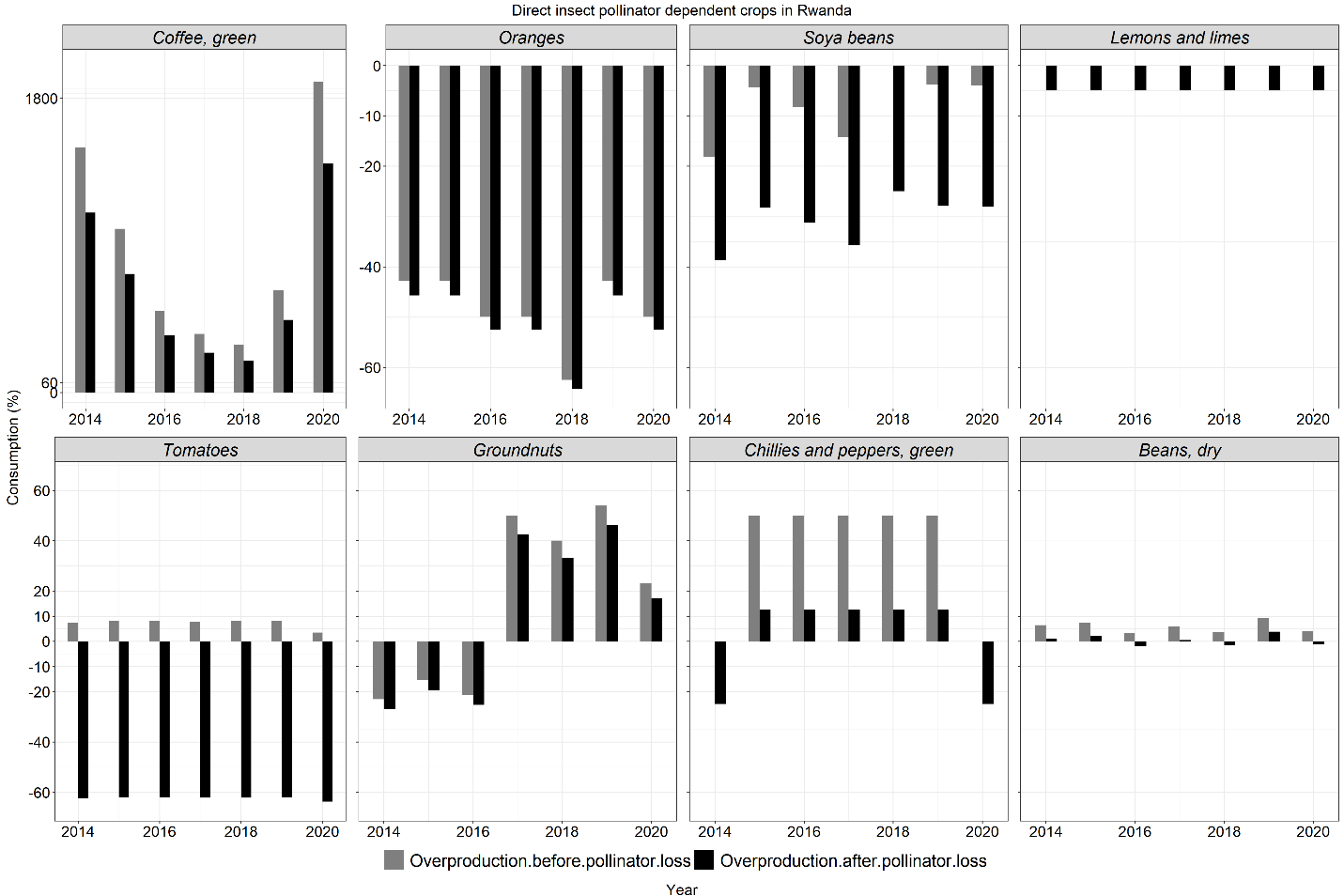


*Figure_2*_*SuppInfo: Overproduction in direct insect pollinator-dependent crops (percentage of consumption) before and after insect pollination loss. Crop production and consumption data for Rwanda were obtained from the Food and Agricultural Organization website* (FAO, 2023)*. Overproduction before (grey bars) and after (black bars), the complete loss of insect pollinators was calculated and represented per crop. Data analysis and visualization were done using the R platform (R version 4.2.2 Development Core Team, 2022) and suitable packages.*


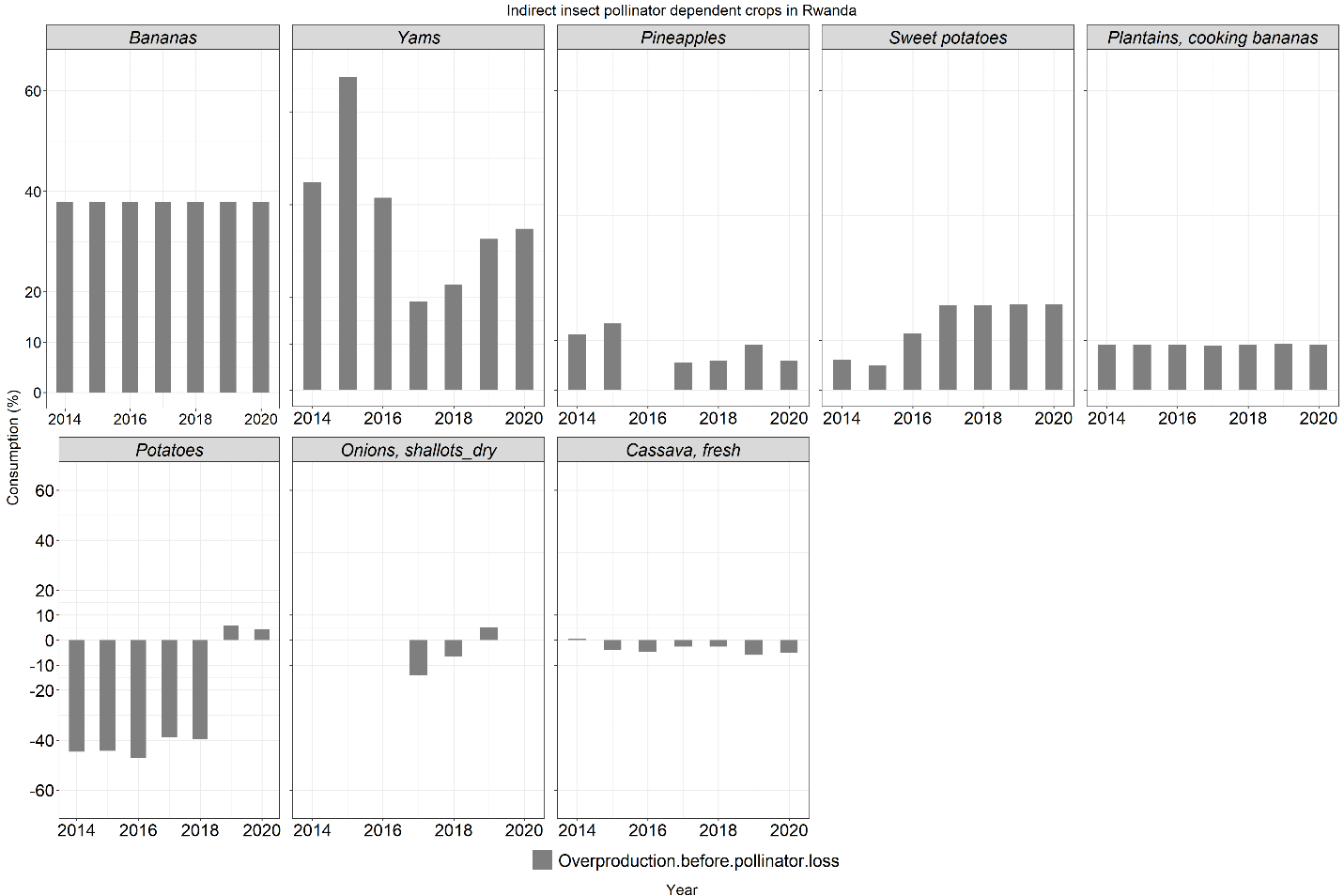


*Figure_3*_*SuppInfo: Overproduction in indirect insect pollinator-dependent crops (percentage of consumption). Crop production and consumption data for Rwanda were obtained from the Food and Agricultural Organization website* (FAO, 2023)*. Overproduction was calculated and represented per crop. Data analysis and visualization were done using the R platform (R version 4.2.2 Development Core Team, 2022) and suitable packages.*


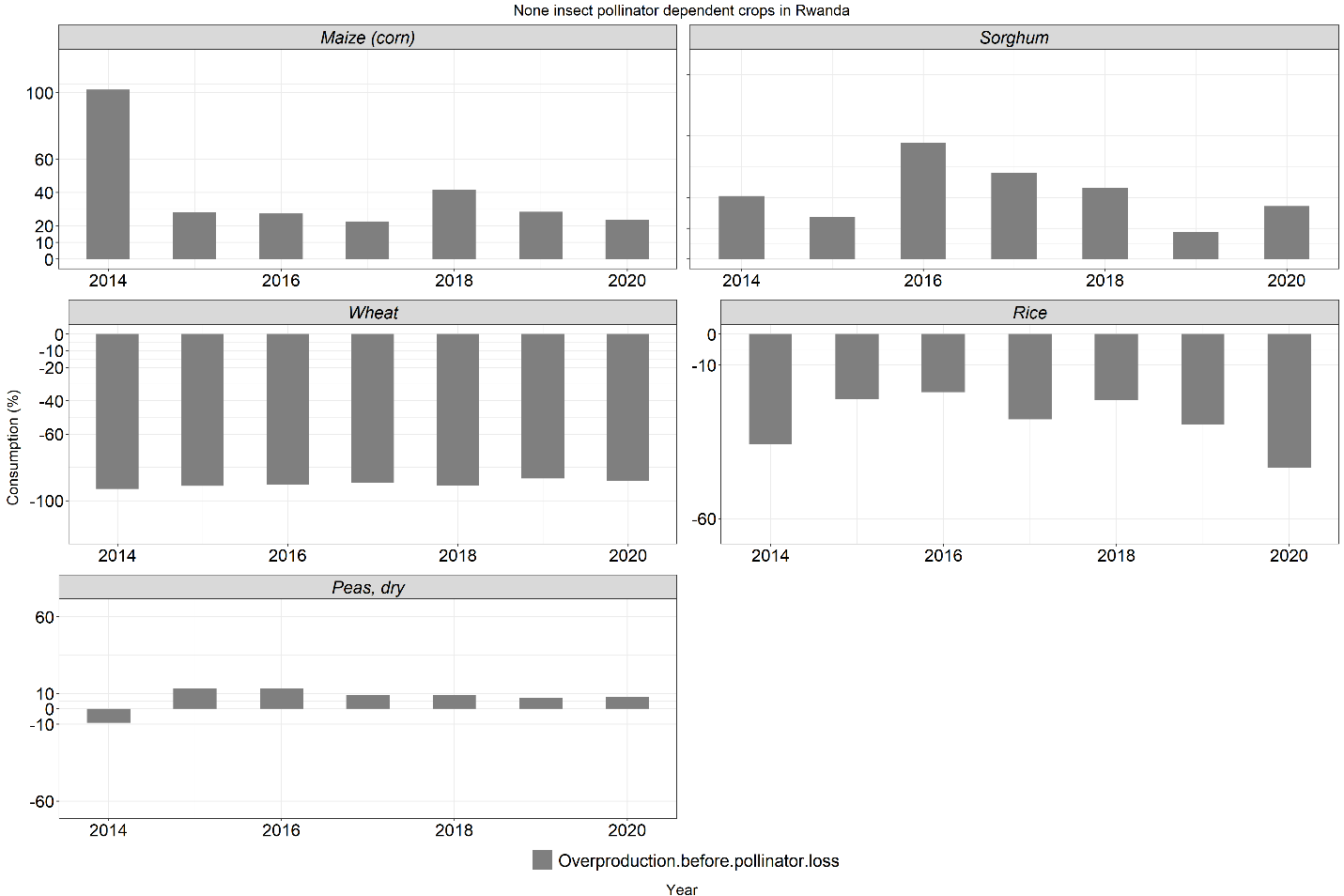


*Figure_4*_*SuppInfo: Overproduction in none-insect pollinator-dependent crops (percentage of consumption). Crop production and consumption data for Rwanda were obtained from the Food and Agricultural Organization website* (FAO, 2023)*. Overproduction was calculated and represented per crop. Data analysis and visualization were done using the R platform (R version 4.2.2 Development Core Team, 2022) and suitable packages.*
